# Tissue-Specific Regulation of Gma-miR396 Family on Coordinating Development and Low Water Availability Responses

**DOI:** 10.3389/fpls.2017.01112

**Published:** 2017-06-26

**Authors:** Weican Liu, Yonggang Zhou, Xiaowei Li, Xingchao Wang, Yuanyuan Dong, Nan Wang, Xiuming Liu, Huan Chen, Na Yao, Xiyan Cui, Aysha Jameel, Fawei Wang, Haiyan Li

**Affiliations:** Engineering Research Center of the Chinese Ministry of Education for Bioreactor and Pharmaceutical Development, College of Life Sciences, Jilin Agricultural UniversityChangchun, China

**Keywords:** *Arabidopsis*, development, drought, *GRF*, low water availability, miR396, soybean

## Abstract

Previously, it was reported that miR396s interact with growth-regulating factors (*GRFs*) to modulate plant growth, development, and stress resistance. In soybean, 11 gma-miR396 precursors (Pre-miR396a–k) were found, and 24 *GmGRFs* were predicted as targets of seven mature gma-miR396s (gma-miR396a/b/c/e/h/i/k). To explore the roles of the miR396–*GRF* module in low water availability response of soybean, we analyzed the expression of Pre-miR396a–k, and found that Pre-miR396a/i/bdgk/e/h were up-regulated in leaves and down-regulated in roots; on the contrary, G*mGRF5/6/7/8/15/17/21* were down-regulated in leaves and *GmGRF1/2/17/18/19/20/21/22/23/24* were up-regulated in roots of low water potential stressed soybean. Any one of gma-miR396a/b/c/e/h/i/k was able to interact with 20 *GmGRFs* (*GmGRF1/2/6–11/13–24*), confirming that this module represents a multi-to-multi network interaction. We generated *Arabidopsis* plants over-expressing each of the 11 gma-miR396 precursors (Pre-miR396a–k), and seven of them (miR396a/b/c/e/h/i/k-OE transgenic *Arabidopsis*) showed altered development. The low water availability of miR396a/b/c/e/h/i/k-OE was enhanced in leaves but reduced in seeds and roots. Contrary to previous reports, miR396a/b/c/i-OE seedlings showed lower survival rate than WT when recovering after rewatering under soil drying. In general, we believe our findings are valuable to understand the role of gma-miR396 family in coordinating development and low water availability responses, and can provide potential strategies and directions for soybean breeding programs to improve seed yield and plant drought tolerance.

## Introduction

MiR396 is a conserved gene family that is found in many plant species, and some *GRF*-family genes are generally recognized as its target genes. MiR396 normally target a conserved domain of *GRF*, the WRC (Trp, Arg, Cys) domain, which contains a functional nuclear localization signal and a putative zinc finger motif (Van der Knaap et al., 2000). The miR396–*GRF* module plays regulatory roles in plant growth and development, and in the responses to various environmental stresses (Omidbakhshfard et al., 2015).

There is some experimental evidence that the miR396-*GRF* module plays significant regulatory roles in plant growth and development (Omidbakhshfard et al., 2015). Yang et al. (2009) reported that ath-miR396a over-expressed in tobacco led to a dwarfed phenotype by targeting *GRF*s, and some *GRFs* were proved to play a role in regulating stem elongation (Kim et al., 2003; Kuijt et al., 2014). Bazin et al. (2013) reported that roots of transgenic *Medicago truncatula* plants over-expressing the mtr-miR396b precursor showed decreased *MtGRFs* expression and reduced growth, but mtr-miR396 inactivation led to increased *MtGRFs* expression and greater root biomass. Rodriguez et al. (2010) reported that transgenic *Arabidopsis* over-expressing ath-miR396b exhibited small leaves, but accumulation of *rGRF2* led to bigger leaves. Wang et al. (2011) and Das Gupta and Nath (2015) suggested that miR396-targeted *AtGRF* transcription factors are required to establish leaf polarity. In addition, Liu et al. (2014) suggested that osa-miR396d regulates the expression of *OsGRFs,* which play roles in controlling floret and spikelet development in rice. However, there have been relatively few reports on the characteristics and functions of the miR396–*GRF* module in crop plants, the families of genes involved in the multi-to-multi miR396–*GRF* network module, and the roles of the different families of genes in plant growth and development.

Several studies have shown that the miR396–*GRF* module is involved in the responses to various biotic and abiotic stresses, including drought, salt, alkali, UV-B radiation, osmotic stresses, and pathogen infection (Gao et al., 2010; Kim et al., 2012; Casadevall et al., 2013; Chen et al., 2015). Previous reports have indicated that miR396-mediated *GRF* regulation affects the low water availability of plants. For example, Liu et al. (2009) and Yang et al. (2009) reported that nta-miR396a and ath-miR396a/b were up-regulated by water deficit. Over-expression of ath-miR396a/b in *Arabidopsis* or tobacco led to down-regulation of *AtGRFs* or *NtGRF*-like genes, respectively, and the transgenic plants showed increased drought tolerance, possibly because of their narrower leaves with a lower stomatal density and reduced water loss rate. Chen et al. (2015) reported that sp-miR396a transcript levels were up-regulated under low water potential stress. Over-expression of sp-miR396a in tobacco led to the down-regulation of four sp-miR396a target genes, *NtGRF1*, *NtGRF3*, *NtGRF7* and *NtGRF8*. The transgenic tobacco plants showed enhanced drought tolerance, and a physiological analysis indicated that sp-miR396a over-expression enhanced osmotic regulation and decreased the production of reactive oxygen species (ROS) in the leaves. These properties suggest that miR396-based genetic modifications have the potential to improve the drought tolerance of plants. However, previous studies have focused only on the leaves of miR396-over-expressing transgenic plants, and none has focused on how other tissues of these transgenic plants respond to low water availability. Therefore, further studies are required to explore the characteristics and functions of the miR396–*GRF* module in the whole plant during the low water availability response.

Soybean (*Glycine max*) is one of the most important legume crops, and the most drought-sensitive crop plant in the legume family (Clement et al., 2008). To date, 11 *Glycine max* gma-miR396 precursors (Pre-miR396a–k) have been identified, and 24 *Glycine max GRFs* (*GmGRF1–GmGRF24*) were predicted as the targets of seven gma-miR396a/b/c/e/h/i/k. These miR396s and *GmGRFs* were identified from sequencing data and annotated by an automated computational analysis (Schmutz et al., 2010; Kulcheski et al., 2011), but their functions have not been confirmed experimentally. In this study, we confirmed that 20 *GmGRFs* are targeted by seven gma-miR396s (gma-miR396a/b/c/e/h/i/k) in soybean using two experimental methods. We verified the function of the gma-miR396 gene family by over-expressing Pre-miR396a–k in *Arabidop*sis (miR396a/b/c/d/e/f/g/h/i/k-OE), seven gma-miR396 precursors (Pre-miR396a/b/c/e/h/i/k) affected the development of miR396a/b/c/e/h/i/k-OE transgenic *Arabidopsis* plants, which exhibited small leaves, short roots, and decreased seed yield. Interestingly, Pre-miR396a/i/bdgk/e/h showed opposite regulation responses in leaves and roots of low water potential stressed soybean. The low water availability of miR396a/b/c/e/h/i/k-OE transgenic plants differed among tissues, enhanced in the small leaves, but reduced in the short roots and transgenic seeds. Contrary to previous reports, miR396a/b/c/i-OE transgenic seedlings in soil drying response displayed more lower survival rate than that of wild type (WT). Together, these data contribute to our understanding of the tissue-specific regulation of the gma-miR396 family in coordinating development and low water availability responses. This information provides directions for soybean breeding programs to improve seed yield and drought tolerance.

## Materials and Methods

### Sequence Analysis and Functional Prediction of Gma-miR396s and Their Targets

*Glycine max* miR396 family gene sequences were obtained from miRBase Release 21^1^. The target genes in *Arabidopsis* (TAIR 10) and *Glycine max* (*Wm82.a2.v1*) were predicted by psRNA Target^2^ with an expectation score of ≤3. Putative targets were retrieved from Phytozome 10.1^3^ and *GRF* family genes were obtained from PLAZA: Comparative Genomics in Plants^4^. Sequences were aligned using ClustalX ver. 2.0.9.

### Soybean Seedling Cultivation and Low Water Potential Stress Treatment

Seeds of the soybean cultivar ‘*Williams 82’* were treated with ethanol for 10 min and then rinsed several times with sterile distilled water. The seeds were cultured in Hoagland’s nutrient solution at 23°C under a 16-h light/8-h dark photoperiod (80 μmol m^-2^ s^-1^ photon flux density) with 50% relative humidity. When the first unifoliate leaves began to open, 8% PEG8000 treatments were initiated, and untreated plants served as controls. The seedlings were kept in low water potential conditions for 3, 6, 12, 48, and 96 h, and leaf and root samples were collected at each time point. All samples were immediately frozen in liquid nitrogen and stored at -80°C until RT-qPCR analysis. Supplementary Figure S1 shows the morphological changes in leaves and roots of soybean seedlings subjected to 96 h of low water potential stress.

### RT-qPCR Analysis

The RT-qPCR assays to quantify the transcript levels of *GmGRFs* and gma-miR396 precursors (Pre-miR396a∼k) were performed according to MIQE guidelines (Bustin et al., 2009). The RT-qPCR method to monitor the expression of microRNA precursors was according to Schmittgen et al. (2004) reports. Total RNA was extracted using RNAiso Plus (Takara, Otsu, Japan). Treatment with DNase, and cDNA synthesis with an RT primer mix Oligo dT and Random 6 mers, were performed using the PrimeScript^TM^ RT Reagent Kit with gDNA Eraser-Perfect Real Time (Takara). The RT-qPCR analysis were performed in 96-well blocks using an Applied Mx3000P Real-Time Thermocycler (Stratagene, La Jolla, CA, United States). The reaction mixtures were prepared using the SYBR Premix Ex Taq II kit (Takara). Suitable reference genes for normalization were evaluated by GeNorm (Vandesompele et al., 2002), and NormFinder (Andersen et al., 2004) was used to identify the combination of reference genes to normalize gene expression levels in soybean. Primer sequences are listed in Supplementary Table S1. The relative amounts of the amplification products were calculated by the 2^-ΔΔCt^ method.

### Verification of Interaction between Gma-miR396s and *GmGRF*-Family Genes

We verified the cleavage site of gma-miR396 on target *GmGRF* transcripts using a modified 5′-RACE method. Briefly, 5 μg total RNA from soybean leaves and roots was ligated to the 5′ RACE adapter and then transcribed into cDNA using the GeneRacer^TM^ (RLM-RACE) kit (Invitrogen, Carlsbad, CA, United States). The 5′-end of each specific transcript was amplified by nested PCR using two inner primers included in the kit and two gene-specific primers (see Supplementary Table S1). The PCR products were cloned into pEASY-T1 (TransGen, Beijing, China) and then sequenced.

We also verified the interaction between gma-miR396 and *GmGRFs* by transient expression assays with *Arabidopsis* mesophyll protoplasts. Since the interaction sites of gma-miR396 and *GmGRFs* are conserved (Supplementary Figure S2), the 21-bp cleavage site sequence CGTTCAAGAAAGCCTGTGGAA was inserted into the HBT-sGFP(S65T)-NOS vector (Chiu et al., 1996) to construct a universal *GmGRF* interaction vector (HBT-sGFP(S65T)-NOS-*GRF*). A 21-bp synonymous mutation sequence CGCTCAAGGAAGCCAGTAGAA was used to produce the control vector (HBT-sGFP(S65T)-NOS-*rGRF*). These constructs are illustrated in Supplementary Figure S3. Protoplasts were isolated from leaves of 30-day-old plants of WT, vector-OE, and miR396a/b/c/e/h/i/k-OE *Arabidopsis* plants. Then, DNA-PEG-calcium transfection was performed as described previously (Yoo et al., 2007). Chloroplast and fluorescence signals were observed under an Olympus IX51 inverted microscope (Olympus, Tokyo, Japan).

### Vector Construction and Generation of Transgenic *Arabidopsis*

Each of the 11 gma-miR396 precursors (Pre-miR396a–k, listed in Supplementary Table S2) was ligated between the CaMV 35S promoter and the Nos-terminator in the modified pBasta vector, with the *Bar* gene inserted into the T-DNA as a selection marker gene. The empty pBasta vector served as the control. *Arabidopsis* (*Columbia*) plants were transformed by *Agrobacterium tumefaciens* (EHA105 strain) using the floral-dip method (Clough and Bent, 1998).

### *Arabidopsis* Growth Conditions and Low Water Availability Stress Treatment

*Arabidopsis* seeds were imbibed for 2 days at 4°C in the dark and then surface-sterilized and sown on budding medium (MS + 2% w/v sugars + 0.8% w/v agar) and with 300 mM D-mannitol for low water potential stress. The seed germination rate (%) and seedling survival rate (%) were analyzed. To analyze the responses of transgenic *Arabidopsis* roots to low water potential stress, 5-day-old seedlings grown on budding medium were transferred into square plastic dishes containing rooting medium (1/2 MS medium +1% w/v sugars + 0.8% w/v agar + 250 mM D-mannitol). The seedlings were cultivated in a tissue culture box under a 16-h light/8-h dark photoperiod at 23°C with 50% relative humidity. *Arabidopsis* seeds were also sown on wet soil, with transgenic *Arabidopsis* and WT grown in the same container. The seedlings were cultivated in a greenhouse under a 12-h light/12-h dark photoperiod at 23°C with 50% relative humidity, and were watered once every 3 days under normal conditions. For soil drying stress treatment, water was withheld from 3-week-old plants grown on soil until the plants wilted, and then watering was resumed.

### Measurement of Morphological Phenotypes

The phenotypes of leaves and roots of *Arabidopsis* and soybean were determined by measuring leaf length (distance from the leaf base to the tip), leaf width (widest distance at the vertical main vein), leaf index (ratio of leaf length to width), leaf area (the area of whole leave), root length (length of main root), lateral root numbers (number of first-class lateral roots), and root diameter (at the middle of the main root). The morphological phenotypes of transgenic *Arabidopsis* were photographed using a camera (Nikon, Tokyo, Japan) or under an inverted microscope (Olympus IX51).

### Analysis of Cells and Stomata in Leaves

Fully expanded fifth leaves of 30-day-old *Arabidopsis* seedlings were fixed overnight in FAA (formalin:acetic acid:70% ethanol, 1:1:8), rehydrated in 70% ethanol for 30 min, and then in 100% ethanol for 30 min before clearing with chloral solution (200 g chloral hydrate, 20 g glycerol, and 50 ml dH_2_O). Leaf palisade cells in the sub-epidermal layer and stomata on the abaxial epidermis were observed using differential interference contrast (DIC) microscopy (TCS-SPE, Leica, Wetzlar, Germany). To analyze stomata density, the epidermis was separated from the leaves of *Arabidopsis* plants, mesophyll cells were removed with a small brush, and then the epidermis was stained with 1% w/v fast green FCF (in 95% ethanol) for 10–20 s, excess dye was rinsed off with water. Palisade cells and stomata at the central region between the mid-vein and the leaf margin were selected for analysis. Paraffin sections were cut using conventional methods to observe the cross section of the main leaf vein. The data were collected and analyzed using Scion Image software.

### Data Analysis

All data shown are means ± SD. Statistical analysis of significant differences and box plots were conducted using SPSS version 17.0 software. To detect significant differences, one-way analysis of variance (ANOVA) was used to compare multiple datasets, the *post hoc* test of LSD was used for ANOVAs; and Student’s *t*-test was used to compare two datasets.

## Results

### Tissue-Specific Regulation of MiR396–*GRF* Module in Soybean Low Water Availability Response

**FIGURE 1:**
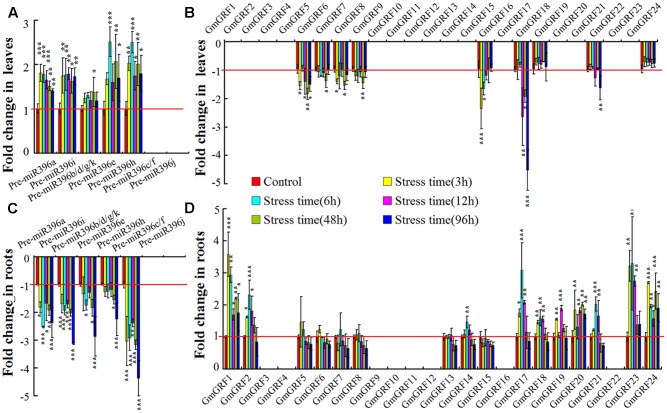
Low water potential stress induced tissue-specific expression of miR396–*GRF* module. **(A,B)** Expression analysis of gma-miR396 precursors in leaves and roots of low water potential stressed soybean. Expression of each gma-miR396 precursor in soybean cultured under normal conditions served as control. **(C,D)** Expression analysis of 24 predicted target *GmGRF* gene*s* of gma-miR396 in leaves and roots of low water potential stressed soybean. Expression of each *GmGRF* gene in soybean cultured under normal conditions served as control. In all panels, values are average of three biological replicates ± SD, different asterisks indicate significant difference applying ANOVA (^∗^*P <* 0.05; ^∗∗^*P <* 0.01; ^∗∗∗^*P <* 0.001). Universal primers were used for expression analysis of Pre-miR396b/d/g/k because of their sequence homology. Because of large deviations in some micro-expression genes data, they were excluded from analysis.

Low water availability status is of most obvious importance in drought stress (Verslues et al., 2006). To analyze the expression of the miR396–*GRF* module during the low water availability response, soybean seedlings were treated with PEG to impose low water potential stress (Supplementary Figure S1). First, we analyzed variations in the expression of gma-miR396 precursors in leaves and roots of low water potential stressed soybean seedlings, Universal primers were used for expression analysis of Pre-miR396b/d/g/k because of their sequence homology. We found that Pre-miR396a, Pre-miR396i, Pre-miR396b/d/g/k, Pre-miR396e, and Pre-miR396h were up-regulated in leaves, but down-regulated in roots; however, the variations in the expression of Pre-miR396c/f and Pre-miR396j in leaves and roots of low water potential stressed soybean seedlings were not detected because they expressed at levels too low to detect (**Figures 1A,B**).

MiRNAs play critical roles by regulating expression levels of target genes, thus, analysis of target genes expression are helpful to understand the function of miRNA. According to prediction, 24 putative *GmGRFs* (*GmGRF1-GmGRF24*) are the targets of 7 gma-miR396 (gma-miR396a/b/c/e/h/i/k), the predicted results were listed in Supplementary Table S3. Therefore, their regulation by gma-miR396 was further analyzed in leaves and roots of low water potential stressed soybean seedlings. In leaves, *GmGRF5/6/7/8/15/17/21* were significantly down-regulated under low water potential stress, *GmGRF18*/*24* were not affected; in roots, *GmGRF1/2/17/18/19/20/21/23/24* were significantly up-regulated, *GmGRF5/6/7/8/13/14/15* were not affected. And *GmGRF1/2/3/4/9/10/11/12/13/14/16/19/20/22/23* in leaves and *GmGRF3/4/9/10/11/12/16/22* in roots were excluded from analysis because their expressed at levels too low to detect (**Figures 1C,D**). Overall, the results showed that, in soybean leaves, low water potential stress up-regulated Pre-miR396a/i/bdgk/e/h and down-regulated *GmGRF5/6/7/8/15/17/21*; in soybean roots, low water potential stress down-regulated Pre-miR396a/i/bdgk/e/h and up-regulated *GmGRF1/2/17/18/19/20/21/23/24*. Excluding genes that were not affected and too low to detect, our results suggested that the miR396–*GRF* module displays tissue-specific regulation in leaves and roots of soybean seedlings in low water availability response.

### Validation of Multi-to-Multi Network Interaction of MiR396–*GRF* Module in Soybean

Target validation of miRNA is a prerequisite step towards understanding the function of miRNA. *GmGRF* gene family contained 26 members (including 55 transcript sequences), among which, 24 *GmGRFs* (including 55 transcript sequences) were predicted as the target genes of 7 gma-miR396 (gma-miR396a/b/c/e/h/i/k) (Supplementary Table S3), and their interaction site was located at the conserved “CGTTCAAGAAAGCCTGTGGAA” sequence (Supplementary Figure S2A) which coded conserved “RSRKPVE” amino acid sequence in the WRC region (Supplementary Figure S2B).

**FIGURE 2:**
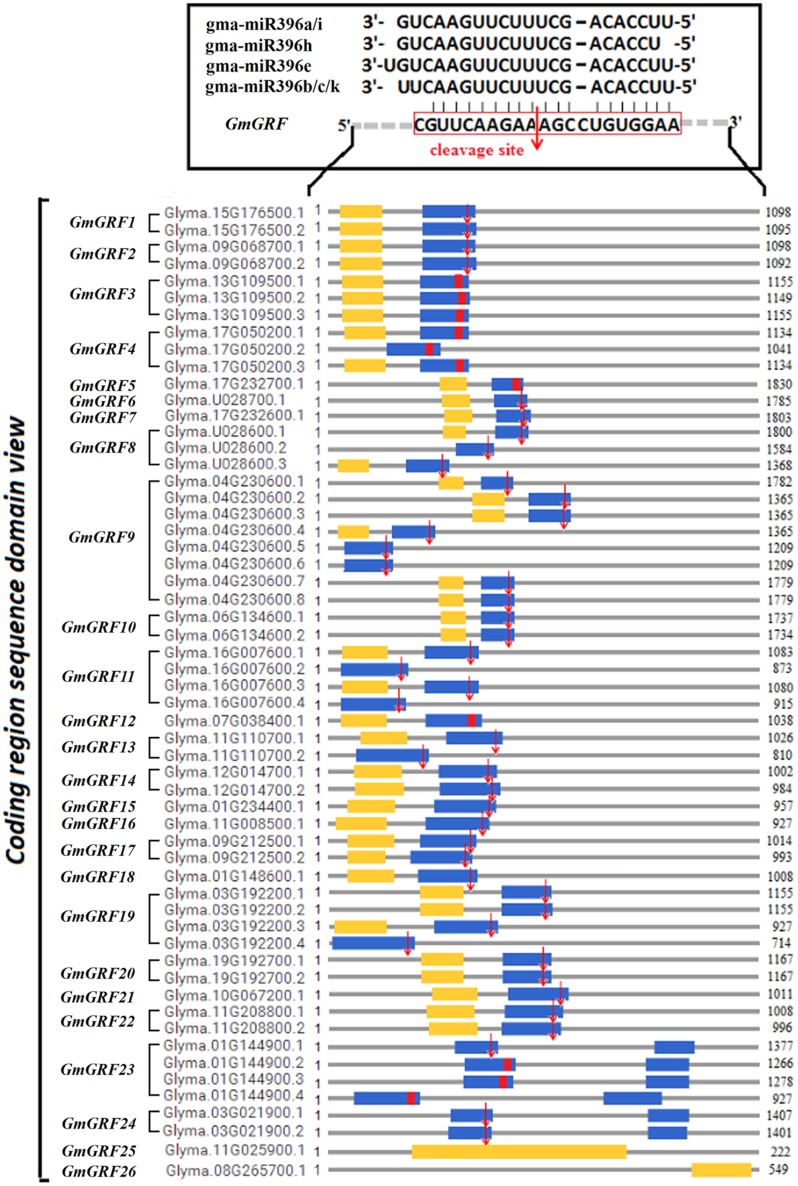
Twenty *GmGRFs* verified to be cleaved by gma-miR396 using 5′RACE. *Glycine max* *GmGRF* family consists of 26 loci (*GmGRF1–GmGRF26*), and 24 *GmGRFs* (*GmGRF1–GmGRF24*) were predicted as target genes of gma-miR396a/b/c/e/h/i/k. Remaining two genes *GmGRF*25 and *GmGRF*26 were not predicted to be target genes of gma-miR396. Figure shows the sequence structure of 57 coding regions: blue squares, WRC conservative coding domain; yellow squares, QLQ conservative coding domain; red squares, predicted conservative cleavage site sequence; red arrows, cleavage sites validated by 5′RACE experiments in this study.

To analyze cleavage of the 24 predicted target *GmGRFs* by the gma-miR396s, we performed a modified 5′-RACE procedure. In this way, we validated that 20 *GmGRFs* (*GmGRF1/2/6–11/13–24*) were cleaved at the same site between CGUUCAAGAA and AGCCUGUGGAA (**Figure 2**). The 5′ termini of mRNA fragments were identified by the cloned 5′-RACE products, which matched to the correct *GmGRF*s and had 5′-ends centered on the miR396-complemented site (Supplementary Figure S4). The cleavage of the other four predicted target *GmGRFs* (*GmGRF3/4/5/12*) was not validated, probably because they expressed at levels too low for detection.

**FIGURE 3:**
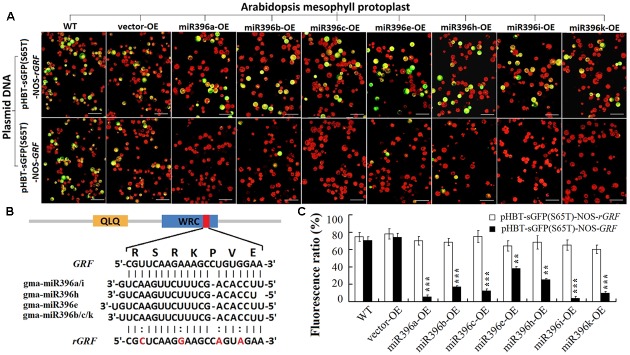
Cleavage of 20 *GmGRFs* by any one of gma-miR396a/b/c/e/h/i/k verified by improved mesophyll protoplast transient transfection technique. **(A)** Plasmid DNA of control vector [HBT-sGFP(S65T)-NOS-*rGRF*] transfected into mesophyll protoplasts (derived from WT, vector-OE, and each of miR396a/b/c/e/h/i/k-OE transgenic *Arabidopsis*) resulted in strong green fluorescence; plasmid DNA of interaction vector [HBT-sGFP(S65T)-NOS-*GRF*] transfected into same mesophyll protoplasts resulted in weaker green fluorescence. (Bar = 50 μm). **(B)** Interaction between gma-miR396a/b/c/e/h/i/k and *GRF/rGRFs*: 20 *GmGRFs* contained predicted cleavage site of gma-miR396a/b/c/e/h/i/k. *rGRF* contained four mutated bases that could not be cleaved by gma-miR396a/b/c/e/h/i/k. **(C)** Fluorescence ratio of mesophyll protoplast cells. Values are average of three replicates ± SD. Asterisks indicate significant difference applying Student’s *t*-test (^∗^*P <* 0.05; ^∗∗^*P <* 0.01; ^∗∗∗^*P <* 0.001).

To determine whether the 20 verified *GmGRF* could interact with any one of gma-miR396a/b/c/e/h/i/k, we used a transient *GFP*-dependent gene expression analysis method in *Arabidopsis* mesophyll protoplasts. When the plasmid DNA of the universal interaction vector (HBT-sGFP(S65T)-NOS-*GRF*) was transfected into *Arabidopsis* mesophyll protoplasts separately isolated from each of the WT/OE-vector and the seven miR396-OE lines (miR396a/b/c/e/h/i/k-OE), the protoplasts derived from the WT/vector showed normal green fluorescence, while those derived from miR396a/b/c/e/h/i/k-OE showed significantly reduced green fluorescence. However, the plasmid DNA of the control vector (HBT-sGFP(S65T)-NOS-*rGRF*) was also transfected into protoplasts isolated from each of the WT/OE-vector and miR396a/b/c/e/h/i/k-OE, and all of them showed normal green fluorescence (**Figure 3A**). The 21-bp cleavage site sequence and its synonymous mutation sequence were illustrated in **Figure 3B**, they were inserted into the HBT-sGFP(S65T)-NOS vector to construct the universal *GmGRF* interaction vector (HBT-sGFP(S65T)-NOS-*GRF*) and the control vector (HBT-sGFP(S65T)-NOS-*rGRF*), respectively. The statistics of the protoplasts fluorescence ratio were illustrated in **Figure 3C**. These results indicated that the conserved interaction site sequence (CGTTCAAGAAAGCCTGTGGAA) could be cleaved by any one of gma-miR396a/b/c/e/h/i/k. That is, the 20 *GmGRFs* (*GmGRF1/2/6-11/13-24*) were able to interact with any one of gma-miR396a/b/c/e/h/i/k. Together, these two datasets confirmed that the seven gma-miR396s (gma-miR396a/b/c/e/h/i/k) and 20 *GmGRFs* (*GmGRF1/2/6-11/13-24*) represent a multi-to-multi network interaction in soybean.

### Effect of Pre-miR396a/b/c/e/h/i/k on Plant Development by Controlling Cell Proliferation and Cell Expansion

**FIGURE 4:**
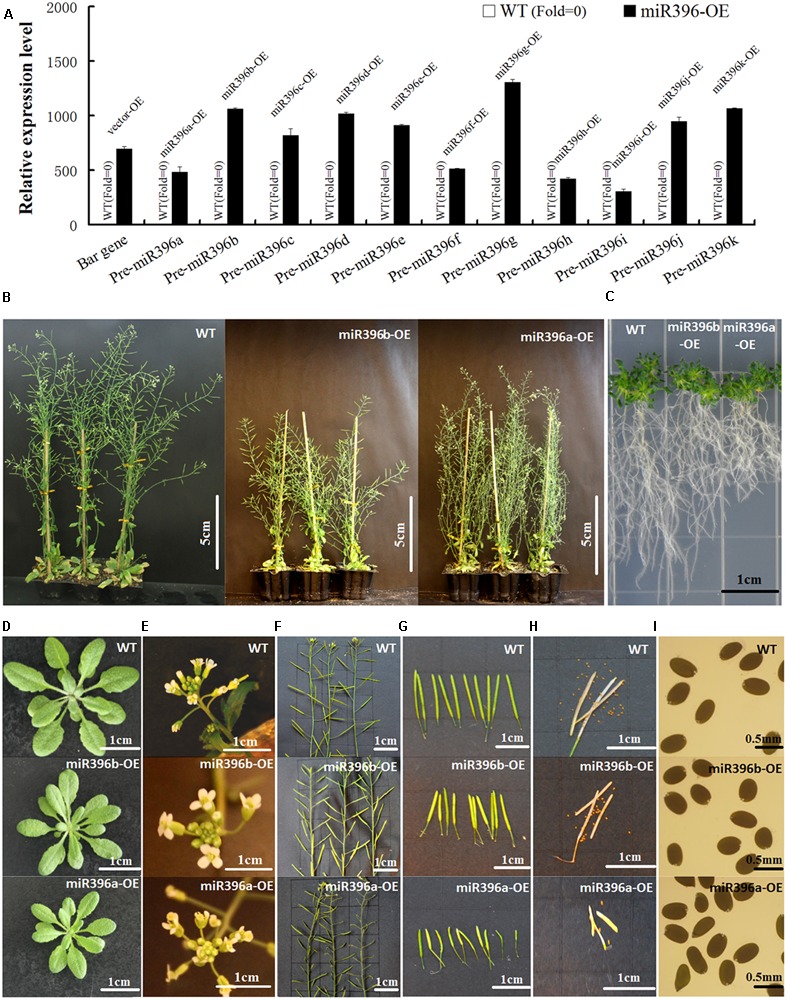
Over-expressing Pre-miR396a/b/c/e/h/i/k in *Arabidopsis* affected tissue development of transgenic plants. **(A)** The expression analysis of bar gene in vector-OE and Pre-miR396a-k in miR396(a-k)-OE. WT was used as control, *Actin*(AT3G46520) was used as reference gene. **(B)** Adult plants; **(C)** Roots on MS + 3% sugars + 0.8% agar; **(D)** Rosette of 30-day-old *Arabidopsis*; **(E)** Inflorescence; **(F)** Stem with siliques; **(G)** Siliques; **(H)** Seeds in one silique; **(I)** Seeds. Compared with WT/vector-OE, miR396a/b/c/e/h/i/k-OE were dwarfed, with short roots, small leaves, and smaller and fewer silique. Only miR396a/i-OE exhibited abnormal silique and flowers. As all miR396a/b/c/e/h/i/k-OE transgenic *Arabidopsis* exhibited similar morphological phenotype, only the WT and miR396a/b-OE plants were shown as representative plants. In addition, miR396d/f/g/j-OE did not display any changes.

To functionally analyze Pre-miR396a–k, we generated transgenic *Arabidopsis* plants constitutively over-expressing each of the 11 gma*-*miR396 family precursors under the control of the CaMV 35S promoter. For each transgenic *Arabidopsis* line (miR396a–k-OE), at least 20 independent transgenic plants were obtained, and the homozygous lines with the highest levels of Pre-miR396 transcripts were used for morphological observations. The results of gene expression analysis showed that bar gene and Pre-miR396a-k in WT have no expression, but bar gene in vector-OE and Pre-miR396a-k in miR396(a-k)-OE transgenic *Arabidopsis* all displayed high over-expression (**Figure 4A**). As miR396d/f/g/j-OE transgenic *Arabidopsis* showed no significant physiological differences compared with WT/vector-OE, so their results on morphological phenotype were omitted. However, other seven of the miR396-OE transgenic *Arabidopsis* lines (miR396a/b/c/e/h/i/k-OE) showed significant phenotypic changes. Compared with WT/vector-OE, miR396a/b/c/e/h/i/k-OE all exhibited dwarf plants (**Figure 4B**), short roots (**Figure 4C**), small leaves (**Figure 4D**), abnormal flowers (**Figure 4E**) and smaller and fewer siliques and seeds (**Figures 4F–I**). It is worth noting that only miR396a/i-OE transgenic *Arabidopsis* displayed abnormal flowers with bent pistil and unfused carpels, which further lead to abnormal siliques. These results indicated that over-expressing Pre-miR396a/b/c/e/h/i/k in *Arabidopsis* could affect tissue development in the transgenic plants.

**FIGURE 5:**
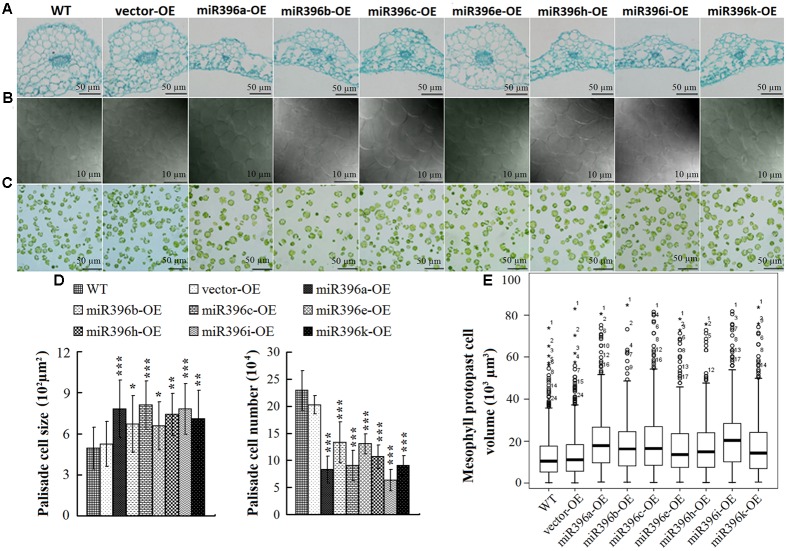
Cell proliferation and expansion in miR396a/b/c/e/h/i/k-OE transgenic *Arabidopsis* leaves. **(A)** Histological sections cut at half-way down main vein. (Bar = 50 μm). **(B)** View of palisade cells at central region between mid-vein and leaf margin (Bar = 10 μm). **(C)** View of mesophyll protoplast. (Bar = 50 μm). **(D)** Palisade cell size and palisade cell number per unit area. Data are average of 30 views ± SD, asterisks indicate significant difference applying ANOVA (^∗^*P <* 0.05; ^∗∗^*P <* 0.01; ^∗∗∗^*P <* 0.001). **(E)** Box-plot chart analysis of mesophyll protoplast cell size (300 cells): box indicates 25 and 75% percentiles, line across the box depicts the median, whiskers represent maximum and minimum values, plus sign represents maximum, or minimum outlier.

To explore the reasons for the changes in leaf dimensions, we analyzed the leaves of miR396a/b/c/e/h/i/k-OE at the cellular level. Comparison of paraffin cross-sections (cut half-way down the leaf main vein) between WT/vector-OE and the transgenic *Arabidopsis* showed that leaf vein cell proliferation was significantly inhibited in the miR396a/b/c/e/h/i/k-OE lines (**Figure 5A**). The leaf palisade cells in the sub-epidermal layer of miR396a/b/c/e/h/i/k-OE were enlarged, but fewer in number compared with those in the WT/vector-OE (**Figures 5B,D**). The box-plot chart showing the cell volume of 300 mesophyll protoplasts clearly showed that the mesophyll protoplast cell volume was significantly larger in miR396a/b/c/e/h/i/k-OE transgenic *Arabidopsis* lines than in the WT/vector-OE (**Figures 5C,E**). Overall, these results indicated that Pre-miR396a/b/c/e/h/i/k regulate leaf development by controlling cell proliferation and cell expansion.

### Tissue-Specific Responses of Low Water Availability in MiR396-OE Transgenic *Arabidopsis* plants

As we already detected tissue-specific regulation of the miR396–*GRF* module in low water potential stressed soybean and we found that Pre-miR396a/b/c/e/h/i/k play practical roles in regulating tissue development, which inspired us to evaluate the low water availability of different tissues of miR396a/b/c/e/h/i/k-OE transgenic *Arabidopsis* plants.

**FIGURE 6:**
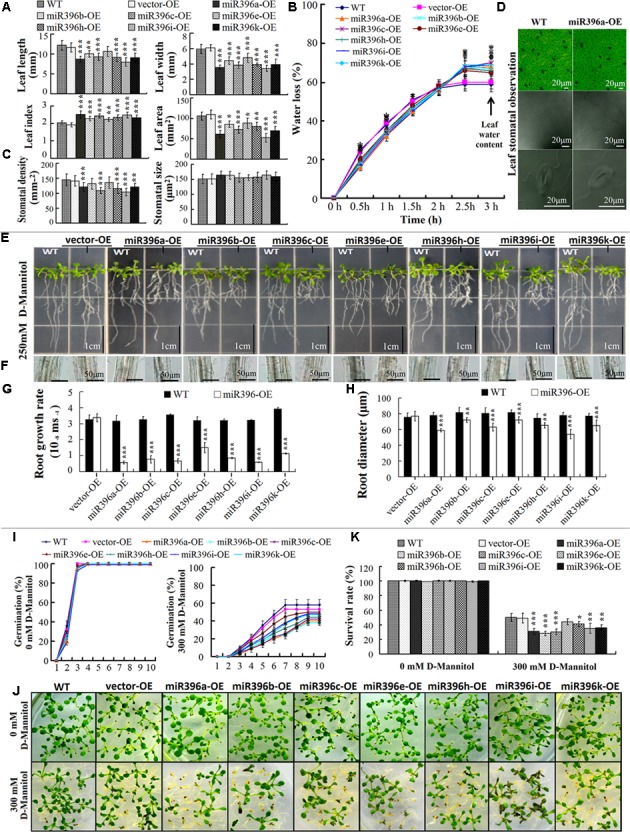
Tissue-specific analysis of miR396a/b/c/e/h/i/k-OE transgenic Arabidopsis response low water availability. **(A)** Analysis for leaf length, width, index, and area; **(B)** Leaf water loss rate and water content; **(C)** Leaf stomata size and density; **(D)** Leaf stomata view (e.g., miR396a-OE). The data from (A) to (C) are average of 30 leaves ± SD, asterisks indicate significant difference applying ANOVA (^∗^*P <* 0.05; ^∗∗^*P <* 0.01; ^∗∗∗^*P <* 0.001). **(E)** Root growth state of *Arabidopsis* cultured on 1/2 MS + 1% sucrose medium + 250 mM D-mannitol; **(F)** Cross section cut at half-way point of main root; **(G)** Root growth rate; **(H)** Root growth diameter. The data for (G) and (H) are average of 30 roots ± SD, asterisks indicate significant difference applying Student’s *t*-test (^∗^*P <* 0.05; ^∗∗^*P <* 0.01; ^∗∗∗^*P <* 0.001). **(J)** Germination rate of *Arabidopsis* seeds in 10 days of cultivation on medium; **(I)** *Arabidopsis* seedlings grown on MS + 2% sugars + 0.8% agar and MS + 2% sugars + 0.8% agar + 300 mM D-mannitol medium for 10 days after germination; **(K)** Survival rate of *Arabidopsis* seeds after 10 days cultivation on medium. The data for **(J,K)** are average of three biological replicates ±SD (200 seeds per replicate). Asterisks indicate significant difference applying ANOVA (^∗^*P <* 0.05; ^∗∗^*P <* 0.01; ^∗∗∗^*P <* 0.001).

To evaluate the low water availability of leaves, the fifth leaves of 30-day-old miR396a/b/c/e/h/i/k-OE transgenic *Arabidopsis* was analyzed. Compared with the WT/vector-OE, the transgenic plants showed reduced leaf length, leaf width, and leaf area, but increased leaf index (length/width ratio) (**Figure 6A**). The transgenic plants had a lower water loss ratio, longer water retention time, and higher leaf water content (**Figure 6B**). Stomata development is known to be affected by cell division and differentiation (Pillitteri and Torii, 2012), so we also analyzed the stomata characteristics of the transgenic plants. The leaf stomata density was significantly lower in miR396a/b/c/e/h/i/k-OE transgenic *Arabidopsis* lines than in the WT/vector-OE, but stomata size was not affected (**Figures 6C,D**). The results indicated that miR396a/b/c/e/h/i/k-OE transgenic *Arabidopsis* lines formed small, narrow leaves with increased the low water availability as a result of their reduced stomata density, which led to greater water holding capacity.

To evaluate the low water availability of roots, we analyzed the root growth characteristics of miR396a/b/c/e/h/i/k-OE transgenic *Arabidopsis* grown on medium supplemented with D-mannitol to impose low water potential stress. The results showed that the main roots of miR396a/b/c/e/h/i/k-OE transgenic *Arabidopsis* were shorter than those of WT/vector-OE on the medium containing 250 mM D-mannitol (**Figure 6E**). Also, the root growth rate was significantly decreased (**Figure 6G**). And the root diameter was smaller in the miR396a/b/c/e/h/i/k-OE seedlings than in the WT/vector-OE on the D-mannitol-containing medium (**Figures 6F,H**). These results indicated that miR396a/b/c/e/h/i/k-OE transgenic *Arabidopsis* roots had decreased the low water availability, as their poor root growth resulted in less water absorption.

To evaluate the low water availability of seeds, we conducted a seed germination assay on medium supplemented with D-mannitol to impose low water potential stress. The seeds of WT/vector-OE and miR396a/b/c/e/h/i/k-OE were sowed on one same plate, their germination rate were calculated very day. All seeds germinated rapidly on the medium without D-mannitol, the germination rate all close to 100% after 3-days. But all seed germination rates increased slowly on medium containing 300 mM D-mannitol, WT/vector-OE after 7-days and miR396a/b/c/e/h/i/k-OE after 9-days close to their maximum, respectively. The germination cycle of miR396a/b/c/e/h/i/k-OE seeds was significantly prolonged, and the germination rate of miR396a/b/c/h/i/k-OE seeds significantly decreased, compared with seeds of the WT/vector-OE (**Figure 6I**). It is worth noting that parts of seeds after germination fail to develop photomorphogenesis, displaying post-germinative growth arrest **(Figure 6J**). At the 10-days after germination, all seeds survival rate showed lower than the germination rate, and survival rate of miR396a/b/c/h/i/k-OE was significantly decreased, compared with seeds of the WT/vector-OE (**Figure 6K**). These results showed that the low water availability of miR396a/b/c/e/h/i/k-OE transgenic *Arabidopsis* seeds was significantly decreased, as indicated by their lower germination potential. Together, these datasets indicated that the low water availability of miR396a/b/c/e/h/i/k-OE transgenic *Arabidopsis* plants differed among the leaves, roots, and seeds.

### MiR396a/b/c/i-OE Transgenic *Arabidopsis* Plants Decreased in Low Water Availability Response

**FIGURE 7:**
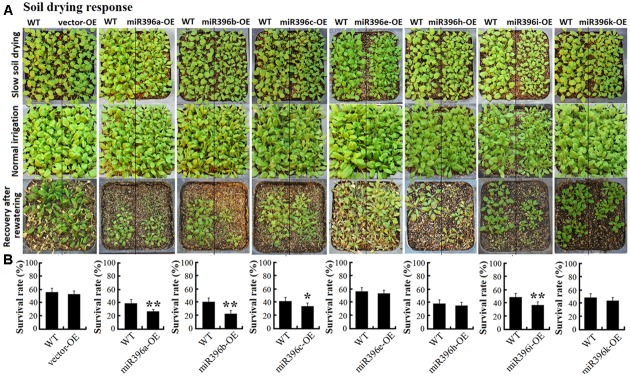
Soil drying response of miR396a/b/c/e/h/i/k-OE transgenic *Arabidopsis* seedlings. **(A)** Photographs of WT, vector-OE, and miR396a/b/c/e/h/i/k-OE *Arabidopsis* for the state of slow soil drying, normal irrigation, and recovery after rewatering. **(B)** Seedling survival rate analysis when *Arabidopsis* were recovery after rewatering. Data are average of five replicates ±SD, asterisks indicate significant difference applying Student’s *t*-test (^∗^*P <* 0.05; ^∗∗^*P <* 0.01; ^∗∗∗^*P <* 0.001).

To evaluate the low water availability of miR396a/b/c/e/h/i/k-OE transgenic *Arabidopsis* seedlings in soil, transgenic *Arabidopsis* and WT seeds were grown side by side, and same number (21:21) seedlings were remained after germination to ensure that the seedlings come out evenly in the same container, to minimize variations arising from differences in the micro-environment. Water was withheld from 3-week-old plants for soil drying treatment. The seedlings were cultivated under soft environment to keep more vegetative growth accompanying slowly soil drying. During the process of soil drying, we found the growth state of miR396a/b/c/e/h/i/k-OE transgenic *Arabidopsis* and WT were always similar, they almostly wilted at same time. Until they wilted (it needed at least one month time), watering was resumed. Parts of seedlings were recovering after rewatering (**Figure 7A**).

The survival rates of transgenic *Arabidopsis* and WT in each container were statistical analyzed when the seedlings recovering after rewatering. As a result, we found the survival rates of the vector-OE were always similar with WT, But the survival rates of miR396a/b/c/e/h/i/k-OE were all decreased to varying degrees. Particularly, the survival rates of miR396a/b/c/i-OE significantly decreased compared with WT under soil drying stress (**Figure 7B**). These results suggested miR396a/b/c/i-OE transgenic *Arabidopsis* plants probably decreased low water availability response.

## Discussion

### Multi-to-Multi Network Interaction of MiR396–*GRF* Module in Soybean

MiRNAs negatively regulate target mRNA gene expression via perfect or near-perfect complementation of the target mRNA. The short sequences of mature miRNAs readily bind to complementary base pairs in multiple target mRNA genes, and many different miRNAs can regulate the same mRNA (Liu and Chen, 2010). That is, miRNAs and their target mRNAs display multi-to-multi network interactions. MiR396 is a conserved gene family in diverse plant species. Previous studies predicted interactions between miR396 and *GRF-*family genes, and these interactions were partially verified at the experimental level in *Arabidopsis* and *Oryza sativa*. In *Arabidopsis*, there are two ath-miR396 loci (ath-miR396a/b), and nine *AtGRF* family genes (*AtGRF*1/2/3/4/5/6/7/8/9), six of which (*AtGRF*1/2/3/7/8/9) were proven to be targets of ath-miR396a/b in 5′-RACE analysis (Jones-Rhoades and Bartel, 2004; Jones-Rhoades et al., 2006). In *O. sativa*, there are eight osa-miR396 loci (osa-miR396a–h) and 13 *OsGRF* family genes, but only some of the predicted *OsGRFs* have been verified as targets of osa-miR396c (Gao et al., 2010; Li et al., 2016).

Soybean has the largest number of miR396/*GRF* family genes identified to date: 11 miR396 loci (gma-miR396a∼k) and 26 *GmGRF* family genes (*GmGRF1–GmGRF26*). Among them, 24 *GmGRFs* (*GmGRF1–GmGRF24*) were predicted to be targets of seven gma-miR396s (gma-miR396a/b/c/e/h/i/k) (Supplementary Table S3). Previous reports identified a few *GmGRF*s as the target genes of gma-miR396 based on their degradation products (Fang et al., 2013; Hu et al., 2013). However, the multi-to-multi interactions of the miR396–*GRF* module in soybean were still unclear, and it was technically difficult to verify the predicted interactions between the seven gma-miR396 and 24 *GmGRFs*. In this study, we used two improved methods, the 5′-RACE technique and the *Arabidopsis* protoplast transient expression, to verify the interaction between the gma-miR396 and *GmGRF* families. The results showed that 20 *GmGRFs* (*GmGRF1/2/6–11/13–24*) can interact with any one of gma-miR396a/b/c/e/h/i/k, and their cleavage sites are conserved between the CGUUCAAGAA and AGCCUGUGGAA sequences. That is, seven gma-miR396 (gma-miR396a/b/c/h/e/i/k) and 20 *GmGRFs* (*GmGRF1/2/6-11/13-24*) in soybean represent a multi-to-multi network interaction.

### MiR396-*GRF* Module Effect on Plant Development

By over-expressing 11 gma-miR396 family precursors (Pre-miR396a–k) in *Arabidopsis*, we verified that Pre-miR396a/b/c/e/h/i/k affect plant development, because the phenotypes of miR396a/b/c/e/h/i/k-OE were dwarf plants, short roots, small leaves, and smaller and fewer siliques. And miR396a/i-OE exhibited abnormal flowers. Previous reports can provide some clues about the regulatory functions of miR396s in plant development based on their target *GRFs*. The *GRF-1* of rice (*OsGRF1*) was shown to play a regulatory role in stem elongation (Knaap et al., 2000; Kim et al., 2003); and silencing of rice *GRF* genes reduced plant height (Kuijt et al., 2014). *Arabidopsis* over-expressing ath-miR396b displayed shorter roots, whereas a *mir396a-1* mutant had longer roots (Bao et al., 2014). The miR396–*GRF* module was suggested to be necessary to regulate the transition of root stem cells into transit-amplifying cells (Rodriguez et al., 2015). Furthermore, our results and those of several previous reports indicate that the miR396–*GRF* module is required to co-ordinate cell division and differentiation during leaf development (Wang et al., 2011). In previous studies, miR396 over-expression markedly decreased the expression of cell cycle-related genes (Liu et al., 2012). Similarly, os-miR396c over-expression decreased rice grain yield, but the synonymous mutation of *OsGRF4* (i.e., disruption of the os-miR396c target) enhanced grain yield (Li et al., 2016). Baucher et al. (2013) and Cao et al. (2016) showed that miR396 targets *GRFs* to control floral organ development, and Liang et al. (2014) reported that miR396 regulates *GRF* to control carpel number and pistil development.

We did not observe any phenotypic changes in miR396d/f/g/j-OE transgenic *Arabidopsis*. In a sequence alignment analysis, the mature miRNA of gma-miR396a/b/c/e/h/i/k(-5P) derived from Pre-miR396a/b/c/e/h/i/k were equivalent or similar to ath-miR396a/b(-5P), the mature miRNA of gma-miR396a/b/c/e/h/i/k(-3P) derived from Pre-miR396a/b/c/e/h/i/k were equivalent or similar to ath-miR396a/b(-3P), and gma-miR396d/f/g/j were dissimilar with them (Supplementary Figure S5A). Ath-miR396a/b-5p have been proved to play roles on development in *Arabidopsis* by target *AtGRF*, but ath-miR396a/b-3p have no functional reports (Omidbakhshfard et al., 2015). According to the target gene prediction of miR396 in soybean and *Arabidopsis*, gma-miR396a/b/c/e/h/i/k-5p same with ath-miR396a/b-5p which interacted with *AtGRF1/2/3/4/7/8/9* (Supplementary Figure S5B). In the present study, our results also confirmed that Pre-miR396a/b/c/e/h/i/k over-expression effect on development in transgenic *Arabidopsis*, the phenotypes of miR396a/b/c/e/h/i/k-OE were similar to ath-miR396a/b OE transgenic *Arabidopsis*. But the special family members Pre-miR396d/f/g/j do not play typical function of miR396 family to effect on development in *Arabidopsis.*

It was also interesting to note that miR396b/c/e/h/k-OE exhibited normal flowers and siliques, and only miR396a/i-OE formed abnormal flowers and silique. This raised the question as to why Pre-miR396b/c/e/h/k and Pre-miR396a/i, which expressed similar mature miRNA sequences that targeted *GRF*-family genes (Supplementary Figure S5), resulted in different phenotypes of floral organs when they were over-expressed in *Arabidopsis*. At present, there is no evidence available to explain their different effects. This should be investigated in future research.

### Tissue-Specific Regulation of Pre-miR396 in Low Water Availability Responses

Plants have evolved defensive strategies to cope with drought stress during evolution (Ferdous et al., 2015). One of the molecular mechanisms is the reprogramming of drought-responsive gene expression by miRNAs (Sunkar et al., 2012). Low water availability status is of most obvious importance in drought stress (Verslues et al., 2006). In previous studies, Liu et al. (2009) found the survived rates of ath-miR396a/b over-expression tansgenic *Arabidopsis* higher than WT after rewatering on soil drying. Yang and Yu (2009) and Chen et al. (2015) found the WT plants leaves were clearly wilted compared to ath-miR396a/sp-miR396-5p over-expression tansgeninc tobacco gown on soil drying. However, we note here that our results are different from those of previous reports. During the process of soil drying, we found the growth state of gma-miR396a/b/c/e/h/i/k OE and WT were always similar, they almost wilted at the same time, and the survival rates of miR396a/b/c/i-OE significantly decreased compared with WT. The inconsistent results probably reflected the high sensitivity of miR396 to subtle differences in soil drying stress conditions, for instance, we cultured the seedlings in one same container, more number of seedlings, more soil drying time, and so on. Another possible reason may be the different precursor-miRNA sequences, which probably led to different efficiency or different mature sequences. This needs further study.

In addition, we found that the low water availability responses of miR396a/b/c/e/h/i/k-OE transgenic *Arabidopsis* differed among tissues. The low water availability responses was enhanced in leaves of miR396a/b/c/e/h/i/k-OE transgenic *Arabidopsis* because the leaves were smaller with lower stomata density, leading to a longer water retention time and higher leaf water content. In contrast, the roots of miR396a/b/c/e/h/i/k-OE transgenic *Arabidopsis* showed reduced low water availability responses because they were shorter and grew poorly under low water potential medium. Similarly, the low water availability was reduced in seeds of miR396a/b/c/e/h/i/k-OE transgenic *Arabidopsis*, as illustrated by their low seed germination potential on low water potential medium. It was interesting that the leaves and roots of miR396a/b/c/i-OE transgenic *Arabidopsis* showed opposite patterns in low water availability, and we found the survival rates of miR396a/b/c/i-OE significantly decreased compared with WT in soil drying response. In most cases, the plant’s first response is to avoid low ψ_w_. In the shorter term, tissue ψ_w_ and water content are maintained close to the unstressed level by increasing water uptake or limiting water loss remain balanced. Such a balance is achieved mainly by stomatal closure. In the longer term, changes in root growth to maximize water uptake are of the greatest importance for plants (Verslues et al., 2006). Our results on low water availability responses among different tissues may provide some evidence to explain why the miR396a/b/c/i-OE transgenic *Arabidopsis* seedlings showed decreased survival rates in the longer-term soil drying response.

We also explored the regulatory mechanism of the miR396–*GRF* regulatory module in soybean in low water availability response. Under low water potential stress, soybean seedlings formed smaller leaves and longer roots (Supplementary Figure S1), both are important adaptive traits when the plants response to low water potential stress (Verslues et al., 2006). Meanwhile, we detected up-regulated expression of Pre-miR396a/i/bdgk/e/h and down-regulated expression of *GmGRF5/6/7/8/15/17/21* in leaves of soybean under low water potential stress; conversely, in roots of drought-stressed soybean, there was down-regulated expression of Pre-miR396a/i/bdgk/e/h and up-regulated expression of *GmGRF1/2/17/18/19/20/21/23/24.* Furthermore, we further verified the function of *Glycine max* miR396-family genes by over-expressing Pre-miR396a–k in *Arabidopsis*, and found miR396a/b/c/e/h/i/k-OE *Arabidopsis* seedlings all exhibited shorter roots and smaller leaves. Taken together, our results suggested that Pre-miR396a/b/c/e/h/i/k are positive regulatory factors in leaves of soybean in low water availability response. They probably inhibit leaf growth by targeting *GmGRFs* to decrease water loss as an adaptation to the low water availability environment. In contrast, Pre-miR396a/b/c/e/h/i/k functioned as negative regulatory factors in roots during the soybean seedling response to low water availability stress, root growth was promoted by enhancing targeted *GRFs* expression to increase the water absorption from soil to adapt to the low water availability environment. Overall, our results illustrate the tissue-specific regulation of the gma-miR396 family in coordinating development and low water availability response. This information will provide potential strategies and directions for soybean breeding programs to improve drought tolerance.

## Author Contributions

HL and FW conceived and designed experiments. WL conducted most of the experiments. YZ participated RLM-RACE experiments. XL, XW, YD, NW, XL, HC, NY, XC, AJ participated data collection. HL and WL wrote the manuscript. All authors read and approved the final manuscript.

## Conflict of Interest Statement

The authors declare that the research was conducted in the absence of any commercial or financial relationships that could be construed as a potential conflict of interest.

## Footnotes

1 http://www.mirbase.org/

2 http://plantgrn.noble.org/psRNATarget/

3 http://phytozome.jgi.doe.gov/pz/portal.html

4 http://bioinformatics.psb.ugent.be/plaza/

## Abbreviations

5′-RACE: RNA ligase-mediated rapid amplification of 5′-cDNA ends
Gma-miR396a-k: Glycine max mature gma-miR396s
*GmGRF*: *Glycine max* growth-regulating factors
*GRFs*: Growth-regulating factors
OE: Over-expression transgenic *Arabidopsis*
RT-qPCR: the reverse transcription quantitative real-time PCR
Pre-miR396a-k: Glycine max gma-miR396 precursors
Vector: Empty plant expression vector
WT: Wild-type *Arabidopsis*.
