# Supplementary material for: Tissue-Specific Regulation of Gma-miR396 Family on Coordinating Development and Low Water Availability Responses
Source: Front Plant Sci. 2017 Jun 26;8:1112. doi: 10.3389/fpls.2017.01112 (PMC5483475; doi:10.3389/fpls.2017.01112)
Supplement: Supplementary file 1 [file Presentation_1.PDF]

Supplementary Material

Tissue-specific Regulation of Gma-miR396 Family on Coordinating Development and Low Water Availability Responses

Weican Liu, Yonggang Zhou, Xiaowei Li, Xingchao Wang, Yuanyuan Dong, Nan Wang, Xiuming Liu, Huan Chen, Na Yao, Fawei Wang*, Haiyan Li*

Engineering Research Center of the Chinese Ministry of Education for Bioreactor and Pharmaceutical Development, College of Life Sciences, Jilin Agricultural University, Changchun, China

***Correspondence:**
Fawei Wang
fw-1980@163.com
Haiyan Li
hyli99@163.com

Table S1. List of primers used in this research.

Primers of gma-miR396 precursors for RT-qPCR analysis				
**Symbol**	**Accession No. (miRBase)**	**Primers**		**Product (bp)**
Pre-miR396a	MI0001785	F：GCTTTCTTGAACTGCATCCAA	R：TCCCACAGCTTTATTGAACCG	94
Pre-miR396i	MI0018680	F：GCTTTCTTGAACTGCATCCAT		94
Pre-miR396b	MI0001786	F：GGTCATGCTTTTCCACAGCTT	R：TTGCCATATTCTCCCACAGC	108
Pre-miR396d	MI0016503			108
Pre-miR396g	MI0017921			108
Pre-miR396k	MI0019764			108
Pre-miR396c	MI0010572	F：CAAGTCCTGTTATGCTTTTCC	R：ACATTTTTCTTAAACTTCAATGC	100
Pre-miR396f	MI0017919			100
Pre-miR396e	MI0016586	F：TGAACTGTGTTGTGAGGCTTC	R：GATTTTCCACAGCTATCTTGAA	92
Pre-miR396h	MI0018679	F：CTTGAACTGTGTTGTGTGAGGT		98
Pre-miR396j	MI0019759	F：GTGTTGTGAGGCTTCTCCAGT		87
**Primers of *GmGRFs* for RT-qPCR analysis**				
**Symbol**	**Accession No. (Phytozome)**	**Primers**		**Product (bp)**
GmGRF1	Glyma.15G176500	F：TCAACAACAACACCATCATCATC	R：TGCAAGAGGTGAGAGAGTGCT	105
GmGRF2	Glyma.09G068700	F：CAATGACAACAACAAACACAAAC	R：TGTCAAGAAACAAGGGAGCC	219
GmGRF3	Glyma.13G109500	F：CTTCCTCTATCATCATTCTTCG	R：TTCAGTCCATACACATACCTG	141

GmGRF4	Glyma.17G050200	F：CTTACCATCATCATCACCATC	R：TGAAATGACAAAGAAACACCAC	109
GmGRF5	Glyma.17G232700	F：GTAGTAGATCAGAAGTATTGCGAA	R：GAGGAAGCATTAGGTGTGTTAG	119
GmGRF6	Glyma.U028700	F：GTGCCTCCAATGAAGCAAATC	R：TTGTCCCACCCATCATCATCA	186
GmGRF7	Glyma.17G232600	F：CTGAAACCAAAGGAGAACAATCC	R：GCAAAGAATCAGAAGTGACAAGC	112
GmGRF8	Glyma.U028600	F：GACTCGTCACTTCTGATTCC	R：GATGACGACGAACGATGGTT	181
GmGRF9	Glyma.04G230600	F：ATTTGAGCTAAGTGGGTGGTT	R：GTTGTTGTTGCTGATGATGTC	109
GmGRF10	Glyma.06G134600	F：CATTTCAGCAAAGTGGGTGTG	R：AATAATGCTTCAGGGCTCTGTT	176
GmGRF11	Glyma.16G007600	F：AGAAATAGGTCTCCGTTCACA	R：CCCACCCAATTGGATGATGT	175
GmGRF12	Glyma.07G038400	F：ATGGACTCTGGGTCTTATTCG	R：GGTTGGTGGTGGCATCATTG	205
GmGRF13	Glyma.11G110700	F：GCTTTCTTTCACCATCCCACG	R：GCAGTACTTGGAGTCTGGGTAT	138
GmGRF14	Glyma.12G014700	F：GAGTTGCCTCTTTTTCTACAT	R：CTTCACGTGTTCTCCCGAT	110
GmGRF15	Glyma.01G234400	F：CAAGGACCTAAATCTGAGGG	R：GAGAATGAGGCAACTCCACT	126
GmGRF16	Glyma.11G008500	F：GGTGGCTGGTCTTCCTGTG	R：AACAATAACTCATGGTGGTAG	110
GmGRF17	Glyma.09G212500	F：TTGATATCGTCTCACCCATCG	R：CAGGCTTTCTTGAACGGTAAC	172
GmGRF18	Glyma.01G148600	F：CAAGAGCCTCCAGTTGATGT	R：GTACTTGGAGTCAGGGTGC	136
GmGRF19	Glyma.03G192200	F：CAATTGTGGTGTAACTGGAGT	R：TCTTCGTCTTCATTCTTGGTC	150
GmGRF20	Glyma.19G192700	F：AGAACCGACGGCAAGAAATG	R：CCTAGAGATCCACCATCACA	155
GmGRF21	Glyma.10G067200	F：GGGATGGCAAATCAAATGGC	R：CCTGAGTTGTTGTTCTGGCTT	112
GmGRF22	Glyma.11G208800	F：CGTTTCTTCTTCACCCTCTT	R：AGACATGGCCATCAGATTTG	145

GmGRF23	Glyma.01G144900	F：GATGGTAGGTGATGTTGGTG	R：GGTTTATTTCTTGTGGAGCAG	146
GmGRF24	Glyma.03G021900	F：CAGTGCGACATCCGTTGTTG	R：AGAAGCTTATTCCAGTAGAGAT	170

Primers of reference genes for RT-qPCR normalization

Symbol	Accession No. (Phytozome)	Primers		Product (bp)
Actin	Glyma.02G091900	F：GACCTTCAACACCCCTGCT	R：GTGGGAGTGCATAACCCTC	143
EF1a	Glyma.19G052400	F：GCTCTTCTTGCTTTCACCCTT	R：TTCCTTCACAATTTCATCATACC	111
Actin	AT3G46520	F：GCTAACCGTGAGAAGATGAC	R：CTAGCATAAAGCGACAGGAC	123

Primers of *GmGRFs* for 5'-RACE analysis

Symbol	Accession No. (Phytozome)	Primers		First round PCR product (bp)	Second round PCR product (bp)
GmGRF1	Glyma.15G176500	3' Primer：GTCATCCATAGAGGAAGCAGAGAAG	3' Nested Primer：GAGCCAGTGTCAAGAAACAAGGGAG	439/433	295
GmGRF2	Glyma.09G068700			439	295
GmGRF3	Glyma.13G109500	3' Primer：GAGTTGCCATGAATCTTCCATTGAGG	3' Nested Primer：GCAGTGTCGAGAAACAAGGGGGCA	481/475	316
GmGRF4	Glyma.17G050200			466	307
GmGRF5	Glyma.17G232700	3' Primer：TGCCTGAGGGAATGTTGTTGTTGG	3' Nested Primer：CTTTGGTTTCAGCTCAAGAGTGGGAG	507	299
GmGRF6	Glyma.U028700		3' Nested Primer：AGGTAGCATGGGAAGGGCAGAGCA	495	275

GmGRF7	Glyma.17G232600		3' Nested Primer：AAGAGTGGGAGGAAGCATGGGAAGT	492	281
GmGRF8	Glyma.U028600		3' Nested Primer：AGAGTGGGAGGCAGCATGGGAAGA	495	289
GmGRF9	Glyma.04G230600	3' Primer：GTGGTATTACAGGAACTAACACTGC	3' Nested Primer：CATCAGAGACTGCCATTGGGAGTTAG	447	389
GmGRF10	Glyma.06G134600	3' Primer：CCCTGGGTTCCTTATTAGCAGAAAC	3' Nested Primer：ATTAGCAGAAACAGAAGCCACGGAGT	335	308
GmGRF11	Glyma.16G007600	3' Primer：AGAAAGCTCTCTCATCCACATCTTC	3' Nested Primer：AGCATCTGGGGGTCTAGAGGAGGTT	437/434	293
GmGRF12	Glyma.07G038400			410	272
GmGRF13	Glyma.11G110700	3' Primer：TGACTCCATTTGGAGAACCTTGTTG	3' Nested Primer：ACTTGGAATCGCATAGGGAATGGAATC	431	230
GmGRF14	Glyma.12G014700	3' Primer：GGATAATCACTATGCAACAGGGAATC	3' Nested Primer：GAAAAGAAGCAGTGTTCACCACCCTC	435	286
GmGRF15	Glyma.01G234400	3' Primer：GCAGATAATCACCCTGCAACATGGA	3' Nested Primer：GATCTCAATGTAGAGAATGAGGCAACT	437	388
GmGRF16	Glyma.11G008500	3' Primer：TCTGTGGCTCCATGTGTAGAACCC	3' Nested Primer：GACAAGAAACTATGTTCCTCAGATTTAGA	341	283

GmGRF17	Glyma.09G212500	3' Primer：GACATTTGAGACCACATGGCATCTG	3' Nested Primer：CTTGGTATTGTCAGCTGAAAGAGTGAG	444	308
GmGRF18	Glyma.01G148600		3' Nested Primer：TCATGTGAAGGTGTGGGAGGGTGG	444	276
GmGRF19	Glyma.03G192200	3' Primer：ATCCACGAGGCAAGTGACGATGC	3' Nested Primer：TGCTCTGGCTCGAGGCTTACATTC	608	454
GmGRF20	Glyma.19G192700	3' Primer：CATGGAAGCCACTTGATTAGACGAG	3' Nested Primer：GACACATCCGAGGAAGCAGCAGC	523	382
GmGRF21	Glyma.10G067200	3' Primer：TGAAGCAGAACACTGGTTGGTGAAG	3' Nested Primer：GTGGAGCTGGTGGTGGATGATCTC	492	442
GmGRF22	Glyma.11G208800		3' Nested Primer：GTGGAGCTGGTGGTGGATGATCTT	492	451
GmGRF23	Glyma.01G144900	3' Primer：CTCTTGGACTTGTTATCTTTCTGGCA	3' Nested Primer：ATCTTAGGAGCAACTGGAGTGGCAAG	420/324/309	322/226/211
GmGRF24	Glyma.03G021900	3' Primer：CTCTTGGACTGGTTATCTTTCTGGCA	3' Nested Primer：CACTGCTGAAGGCAGCTATCTTAGGG	423	339

Table S2. The sequences information of *Glycine max* miR396 gene family.

***Glycine max* miR396 gene family stem-loop sequences**				
**Symbol**	**Accession**	**Sequence**	**Length**	**Genome context**
Pre-miR396a	MI0001785	UCAUGGCUCUCUUUGUAUUCUUCCACAGCUUUCUUGAACUGCAUCCAAAGAGUUCCUUUGCAUGCAUGCCAUGGCACUCUUACUCCCAAAUCUUGUUUUGCGGUUCAAUAAAGCUGUGGGAAGAUACAGAUAGGGUCAAC	140 bp	chr13:26338134-26338273 [-]
Pre-miR396b	MI0001786	CUCAAGUCCUGGUCAUGCUUUUCCACAGCUUUCUUGAACUUCUUAUGCAUCUUAUAUCUCUCCACCUCCAGGAUUUUAAGCCCUAGAAGCUCAAGAAAGCUGUGGGAGAAUAUGGCAAUUCAGGCU	126 bp	chr13:26329931-26330056 [+]
Pre-miR396c	MI0010572	CAACAAGUCCUGUUAUGCUUUUCCACAGCUUUCUUGAACUUCUUAUGCCUAGUGCAAUUAUUGAUGUGGCAUAGAAGUUUAAGAAAAAUGUGGAAAAACAUGUCAAAUCUAGGACUU	117 bp	chr13:43804777-43804893 [+]
Pre-miR396d	MI0016503	GGUCAUGCUUUUCCACAGCUUUCUUGAACUUCUUAUGCAUCUUAUAUCUCUCCACUUCCAGCAUUUUAAGCCCUAGAAGCUCAAGAAAGCUGUGGGAGAAUAUGGCAA	108 bp	chr17:9053049-9053156 [-]
Pre-miR396e	MI0016586	GUGAUCUUCCACAGCUUUCUUGAACUGUGUUGUGAGGCUUCUCUCCAAUGAAGGUUUAUACCCUAUGCAAAAGAAAUUCUAUGAGCACAAUUCAAGAUAGCUGUGGAAAAUCAC	114 bp	chr17:35366545-35366658 [-]

Pre-miR396f	MI0017919	UAGCUUCUUCAGCAUUUCAACUUCCAUGCUUGCUUGAACAAGUCCUGUUAUGCUUUUCCACAGCUUUCUUGAACUUCUUAUGCCUAAUGCAGCUAUUGAUGUGGCAUUGAAGUUUAAGAAAAAUGUGGAAAAACAUGUCAAAUCUAGGACU	151bp	chr15:556691-556841 [-]
Pre-miR396g	MI0017921	AUGCUGUGUGUGUGAGAUCUGAGCUCAAUUUUCCUCUCAAGUCCUGGUCAUGCUUUUCCACAGCUUUCUUGAACUUCUUAUGCAUCUUAUAUCUCUCCACUUCCAGCAUUUUAAGCCCUAGAAGCUCAAGAAAGCUGUGGGAGAAUAUGGC	151bp	chr17:9053051-9053201 [-]
Pre-miR396h	MI0018679	GAAUGGUCUUUUUCGUGAUCUUCCACAGCUUUCUUGAACUGUGUUGUGUGAGGUUUCUCCAAGUGAAGGUUUAAGAUCCCUUAUGCAACAUAAAUUCUUUGAGCACAAUUCAAGAUAGCUGUGGAAAAUCACUGAGAUGAUCUCGUUC	148 bp	chr14:13971419-13971566 [+]
Pre-miR396i	MI0018680	UGGCCCUCUUUGUAUUCUUCCACAGCUUUCUUGAACUGCAUCCAUAGAGUUCCUUUGCAUGCAUGCCAAGGCACUCUUGCUCCCACACCUUGUUUUGCGGUUCAAUAAAGCUGUGGGAAGAUACAGAUAGGGUCA	135 bp	chr17:9044850-9044984 [+]
Pre-miR396j	MI0019759	GGUUUUCGUGAUCUUCCACAGUUUUCUUGAACUGCAUGUGUGUUGUGAGGCUUCUCCAGUGAAGGUUUAUCUCCUAUGCAAGUGCAGAUAUUCUAUGAGCACAAUUCAAGAUAGCUGUGGAAAAUCACUGAGAU	134bp	chr16:31162190-31162323 [-]
Pre-miR396k	MI0019764	UUCCUCUCAAGUCCUGGUCAUGCUUUUCCACAGCUUUCUUGAACUUCUUAUGCAUCUUAUAUCUCUCCACUUCCAGCAUUUUAAGCCCUAGAAGCUCAAGAAAGCUGUGGGAGAAUAUGGCAAUUCAGGCUUUUAAUUG	139bp	chr17:9053033-9053171 [-]

Glycine max **miR396 gene family mature sequences**			
**Symbol**	**Accession**	**Sequence**	**Length**
gma-miR396a	MIMAT0001687	UUCCACAGCUUUCUUGAACUG	21bp
gma-miR396a-3p	MIMAT0020922	UUCAAUAAAGCUGUGGGAAG	20bp
gma-miR396b	MIMAT0001688	UUCCACAGCUUUCUUGAACUU	21bp
gma-miR396b-3p	MIMAT0020923	GCUCAAGAAAGCUGUGGGAGA	21bp
gma-miR396c	MIMAT0010079	UUCCACAGCUUUCUUGAACUU	21bp
gma-miR396d	MIMAT0018262	AAGAAAGCUGUGGGAGAAUAUGGC	24bp
gma-miR396e	MIMAT0018345	UUCCACAGCUUUCUUGAACUGU	22bp
gma-miR396f	MIMAT0021069	AGCUUUCUUGAACUUCUUAUGCCUA	25bp
gma-miR396g	MIMAT0021071	UUCUUGAACUUCUUAUGCAUC	21bp
gma-miR396h	MIMAT0021668	UCCACAGCUUUCUUGAACUG	20bp
gma-miR396i	MIMAT0021669	UUCCACAGCUUUCUUGAACUG	21bp
gma-miR396i-3p	MIMAT0021670	GUUCAAUAAAGCUGUGGGAAG	21bp
gma-miR396j-3p	MIMAT0023214	AUUCAAGAUAGCUGUGGAAAA	21bp
gma-miR396k	MIMAT0032132	UUCCACAGCUUUCUUGAACUU	21bp
gma-miR396k-3p	MIMAT0023219	GCUCAAGAAAGCUGUGGGAGA	21bp

Table S3. Target genes prediction of gma-miR396 nature sequences in *GRF* gene family for soybean and *Arabidopsis*

Symbol	Accession (Phytozome)	Expectation (E)	Inhibition	Target region	Descriptions
**Predicted target growth-regulating factors (*GRFs*) for gma-miR396a, gma-miR396b, gma-miR396c, gma-miR396e, gma-miR396h, gma-miR396i and gma-miR396k in soybean.**					
GmGRF1	Glyma.15G176500.1; Glyma.15G176500.2;	2	Cleavage	CDS	growth-regulating factor 1
GmGRF2	Glyma.09G068700.1; Glyma.09G068700.2;	2	Cleavage	CDS	growth-regulating factor 1
GmGRF3	Glyma.13G109500.1;Glyma.13G109500.2; Glyma.13G109500.3	2	Cleavage	CDS	growth-regulating factor 1
GmGRF4	Glyma.17G050200.1; Glyma.17G050200.2; Glyma.17G050200.3	2	Cleavage	CDS	growth-regulating factor1
GmGRF5	Glyma.17G232700.1	2	Cleavage	CDS	growth-regulating factor 1
GmGRF6	Glyma.U028700.1	2	Cleavage	CDS	growth-regulating factor 6
GmGRF7	Glyma.17G232600.1	2	Cleavage	CDS	growth-regulating factor 6
GmGRF8	Glyma.U028600.1; Glyma.U028600.2; Glyma.U028600.3	2	Cleavage	CDS	growth-regulating factor 7
GmGRF9	Glyma.04G230600.1; Glyma.04G230600.2; Glyma.04G230600.3; Glyma.04G230600.4; Glyma.04G230600.5; Glyma.04G230600.6; Glyma.04G230600.7; Glyma.04G230600.8	2	Cleavage	CDS	growth-regulating factor 7
GmGRF10	Glyma.06G134600.1; Glyma.06G134600.2	2	Cleavage	CDS	growth-regulating factor 7

GmGRF11	Glyma.16G007600.1; Glyma.16G007600.2; Glyma.16G007600.3; Glyma.16G007600.4	2	Cleavage	CDS	growth-regulating factor 5
GmGRF12	Glyma.07G038400.1	2	Cleavage	CDS	growth-regulating factor 5
GmGRF13	Glyma.11G110700.1; Glyma.11G110700.2	2	Cleavage	CDS	growth-regulating factor 5
GmGRF14	Glyma.12G014700.1; Glyma.12G014700.2	2	Cleavage	CDS	growth-regulating factor4
GmGRF15	Glyma.01G234400.1	2	Cleavage	CDS	growth-regulating factor 4
GmGRF16	Glyma.11G008500.1	2	Cleavage	CDS	growth-regulating factor 4
GmGRF17	Glyma.09G212500.1; Glyma.09G212500.2	2	Cleavage	CDS	growth-regulating factor4
GmGRF18	Glyma.01G148600.1	2	Cleavage	CDS	growth-regulating factor 3
GmGRF19	Glyma.03G192200.1; Glyma.03G192200.2; Glyma.03G192200.3; Glyma.03G192200.4	2	Cleavage	CDS	growth-regulating factor 3
GmGRF20	Glyma.19G192700.1; Glyma.19G192700.2	2	Cleavage	CDS	growth-regulating factor 3
GmGRF21	Glyma.10G067200.1	2	Cleavage	CDS	growth-regulating factor 3
GmGRF22	Glyma.11G208800.1 Glyma.11G208800.2	2	Cleavage	CDS	growth-regulating factor 3
GmGRF23	Glyma.01G144900.1; Glyma.01G144900.2; Glyma.01G144900.3; Glyma.01G144900.4	2	Cleavage	CDS	growth-regulating factor 9
GmGRF24	Glyma.03G021900.1; Glyma.03G021900.2	2	Cleavage	CDS	growth-regulating factor 9
GmGRF25	Glyma.11G025900.1	—	—	—	growth-regulating factor
GmGRF26	Glyma.08G265700.1	—	—	—	growth-regulating factor1

Predicted target growth-regulating factors (*GRFs*) for gma-miR396a, gma-miR396b, gma-miR396c, gma-miR396e, gma-miR396h, gma-miR396i and gma-miR396k in *Arabidopsis*.

Symbol	Accession (Phytozome)	Expectation (E)	Inhibition	Target region	Descriptions
AtGRF1	AT2G22840	2	Cleavage	CDS	growth-regulating factor 1
AtGRF2	AT4G37740	2	Cleavage	CDS	growth-regulating factor 2
AtGRF3	AT2G36400	2	Cleavage	CDS	growth-regulating factor 3
AtGRF4	AT3G52910	2	Cleavage	CDS	growth-regulating factor 4
AtGRF5	AT3G13960	—	—	—	growth-regulating factor 5
AtGRF6	AT2G06200	—	—	—	growth-regulating factor 6
AtGRF7	AT5G53660	2	Cleavage	CDS	growth-regulating factor 7
AtGRF8	AT4G24150	2	Cleavage	CDS	growth-regulating factor 8
AtGRF9	AT2G45480	2.5	Cleavage	CDS	growth-regulating factor 9

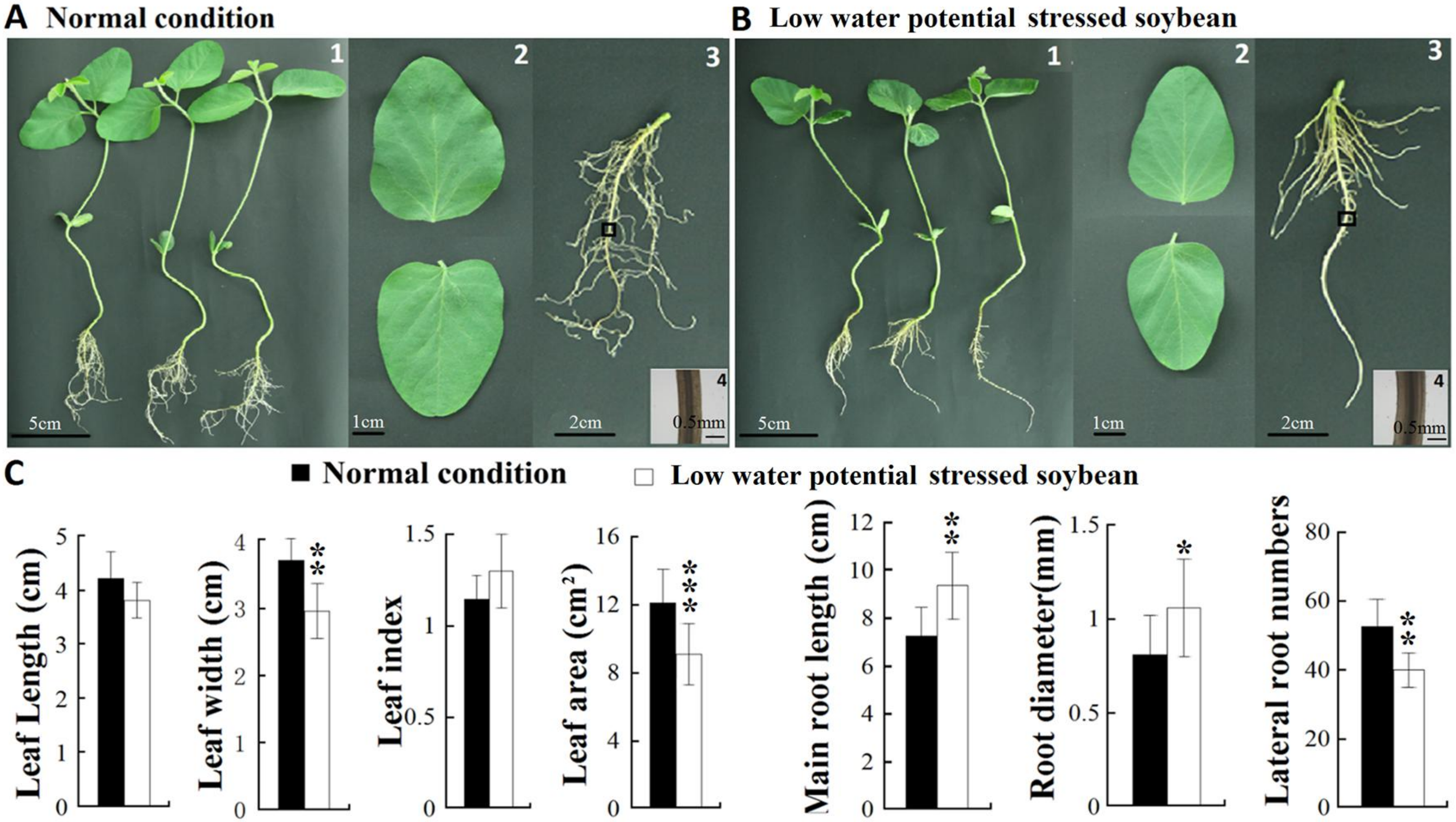

Figure S1. Low water popential stress effect on morphological changes of soybean seedling leaves and roots. (A) Morphological observations of soybean seedlings cultured under normal condition. **(B)** Morphological observations of soybean seedlings cultured on low water popential stress treatment condition lasting for 96h. **(C)** Morphology parameter determination of roots and leaves for soybean seedlings under normal condition and low water popential stress condition. Compared with soybean cultured under normal condition, the low water popential stressed soybean seedlings showed smaller leaves and stronger roots, which displayed smaller width and area, and main roots turn longer with wider root diameter and more lateral roots. It is common to observe inhibition of leaf growth and promotion of root development in plants responding to low water availability. So this indicated soybean seedlings were growing in the direction of adaption to low water availability stress. The data represent ten biological replicates ± SD, asterisks indicate significant differences applying the Student's t test (*****, $P<0.05$; ******, $P<0.01$; *******, $P<0.001$).

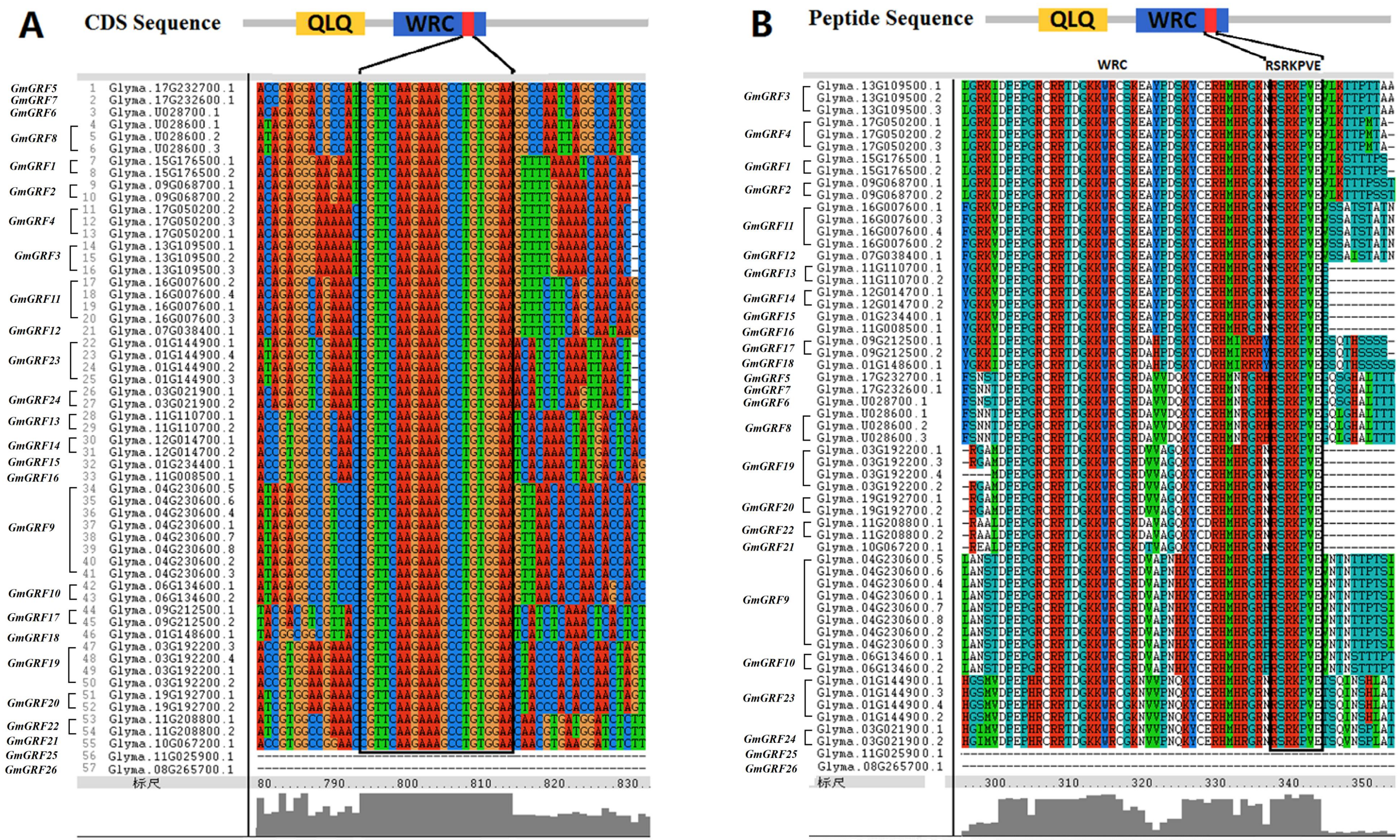

Figure S2. The cleave sites prediction of gma-miR396 on *GmGRF* family genes. (A) The predicted cleave sites of gma-miR396 on nucleotide sequences of *GmGRF* gene family. (B) The predicted cleave sites of gma-miR396 on amino acid sequences of *GmGRF* protein family. The 21bp conserved sequence "CGTTCAAGAAAGCCTGTGGAA" in squares were the cleave sites of gma-miR396 on 24 *GmGRF*s which coded "RSRKPVE" protein sequences. Other two *GmGRFs* were not the predicted targets of gma-miR396.

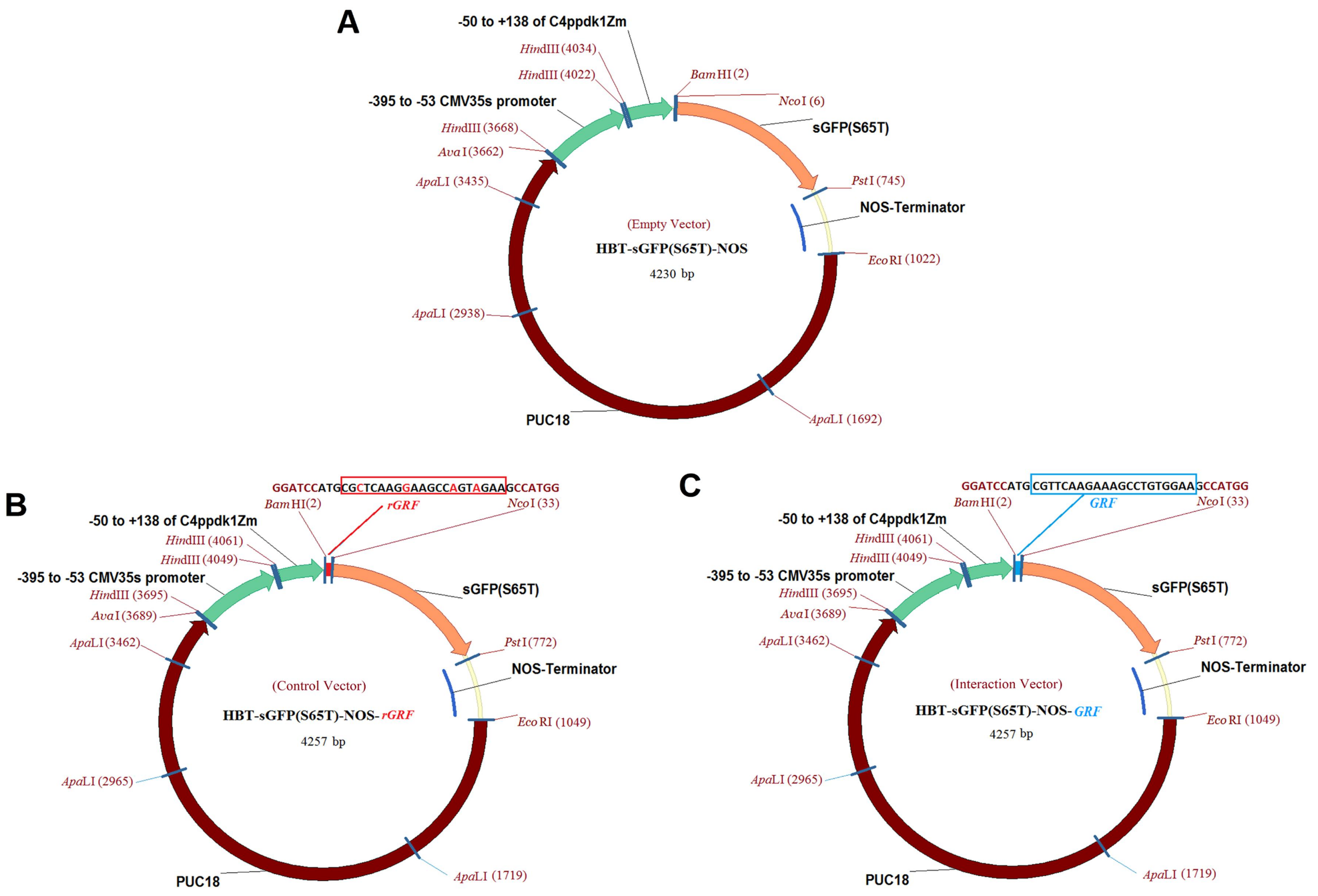

Figure S3. The construction map of vector for validating the interaction between gma-miR396s and *GmGRFs* by *Arabidopsis* mesophyll cells transient transformation. (A) The HBT-sGFP(S65T)-NOS vector was used for vector construction. (B) The 21bp conserved sequence “CGTTCAAGAAAGCCTGTGGAA” of cleavage site was inserted into HBT-sGFP(S65T)-NOS vector to get an universal *GmGRF* interaction vector which named as HBT-sGFP(S65T)-NOS-*GRF*. (C) The 21bp synonymous mutation sequence “CGCTCAAGGAAGCCAGTAGAA” of cleavage site were insert into HBT-sGFP(S65T)-NOS vector as control vector which named as HBT-sGFP(S65T)-NOS-*rGRF*.

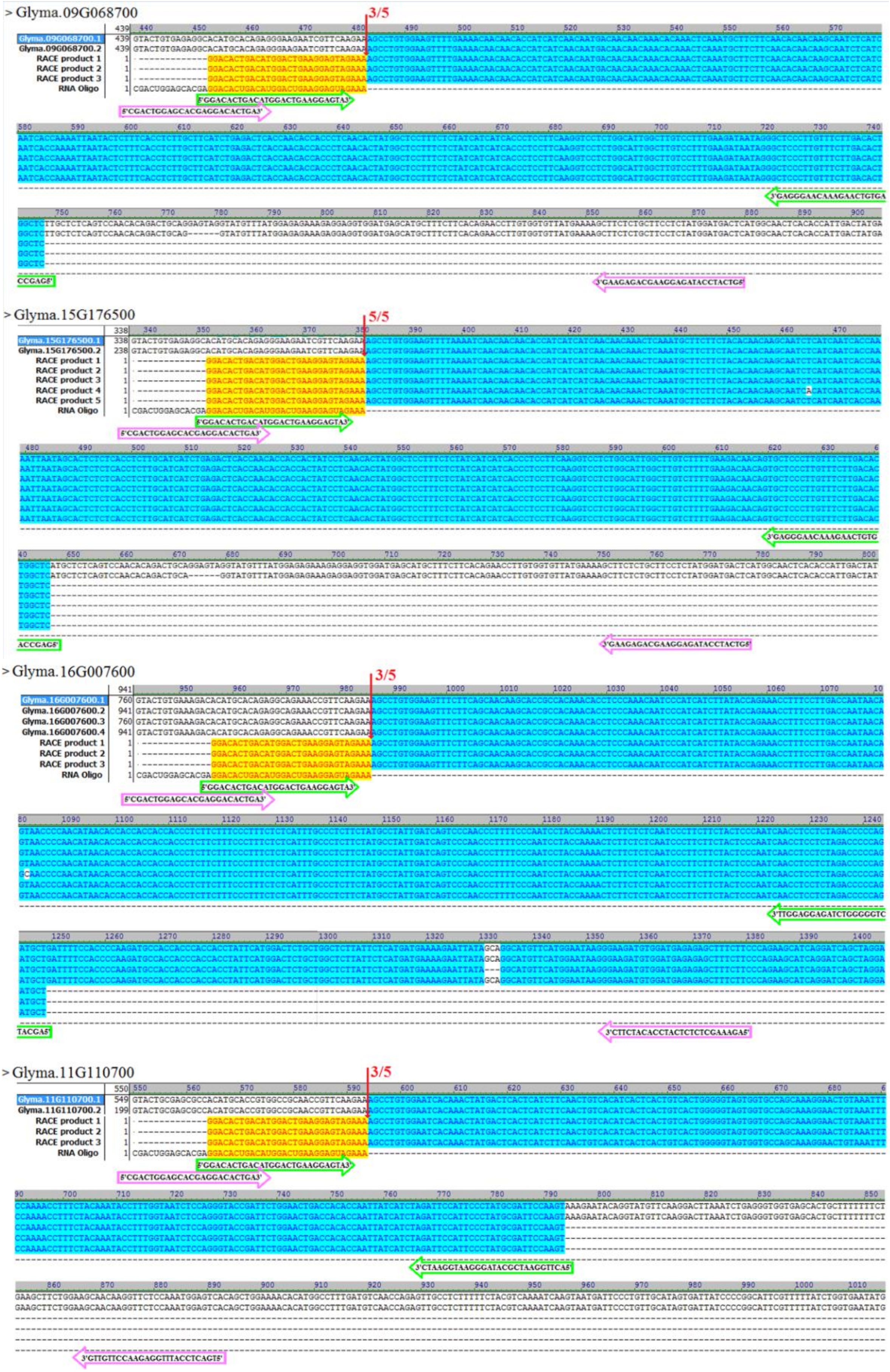

Continuing next

> Glyma.12G014700

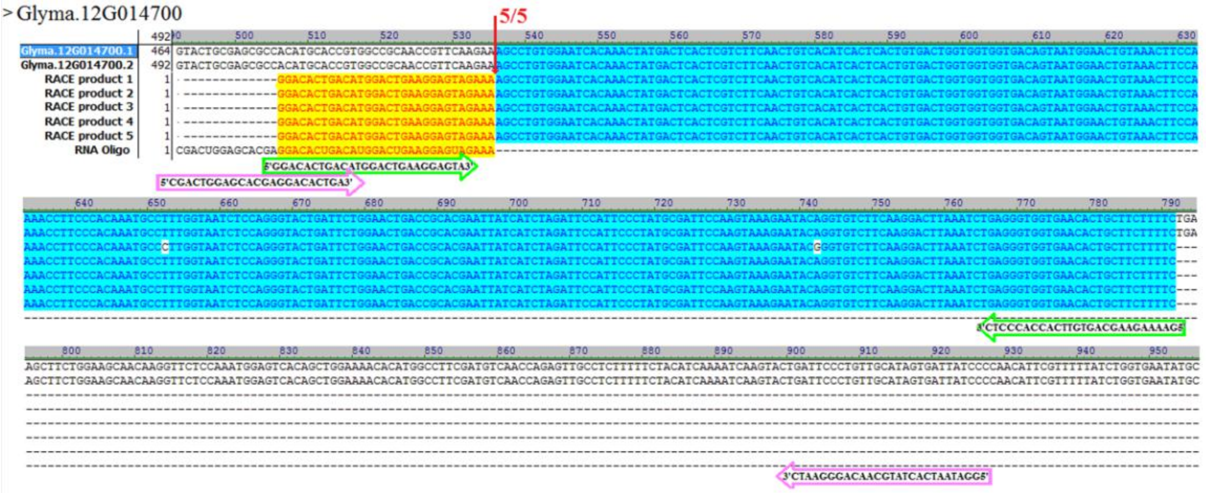

> Glyma. 04G230600

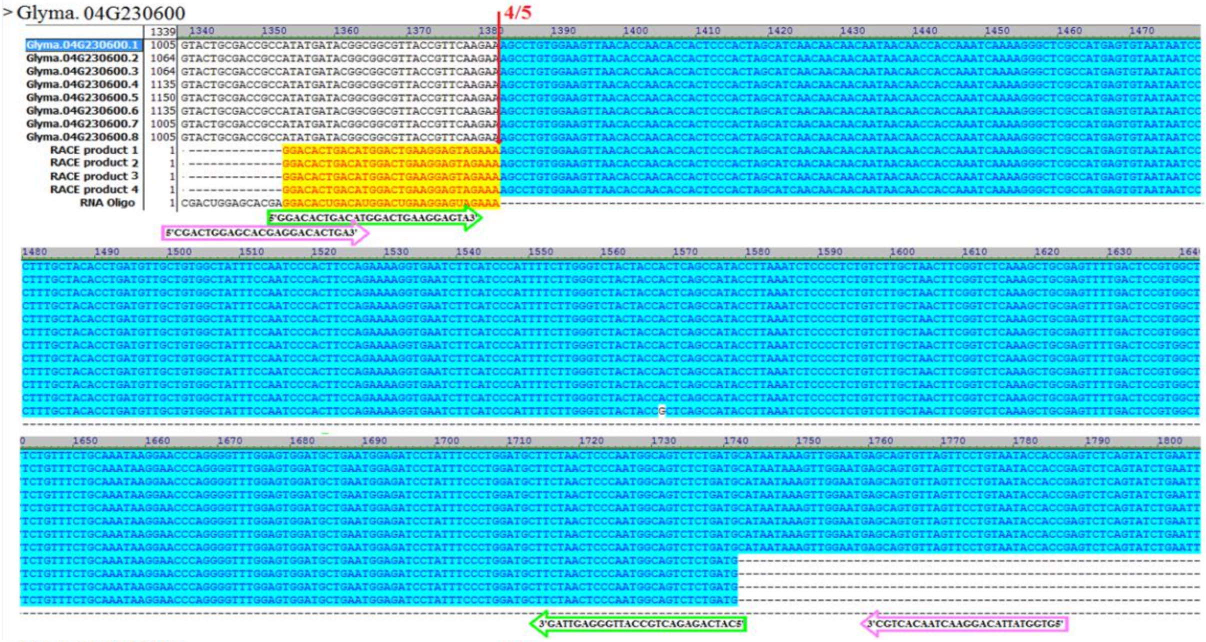

> Glyma.03G192200

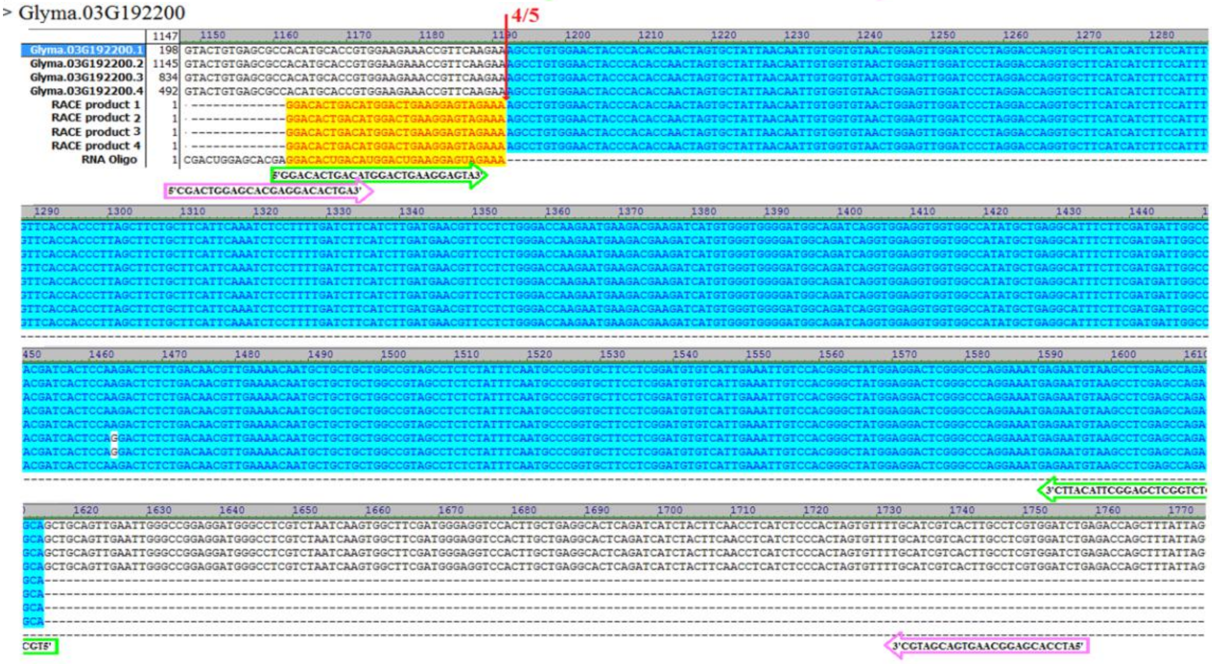

Continuing next

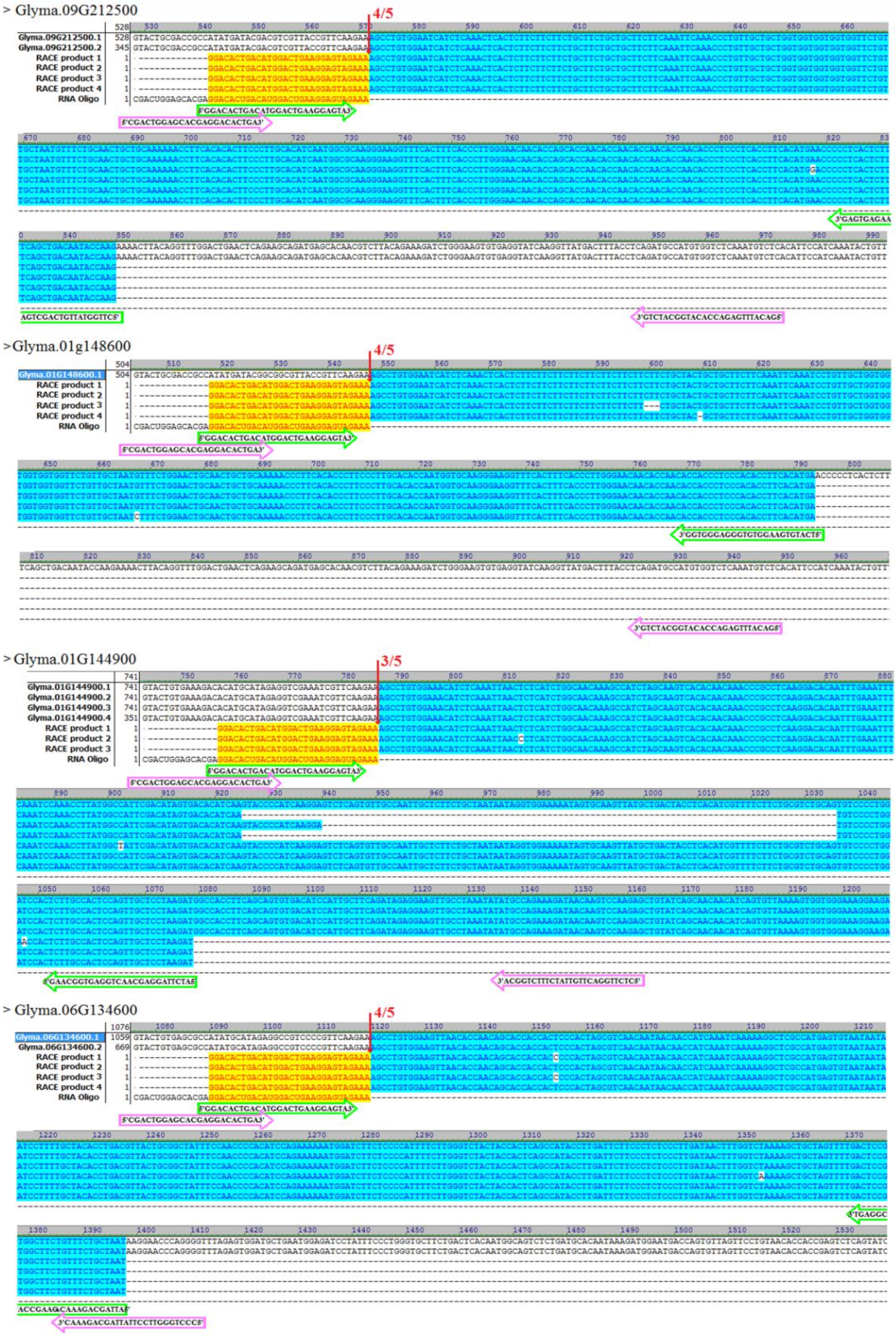

Continuing next

> Glyma.19G192700

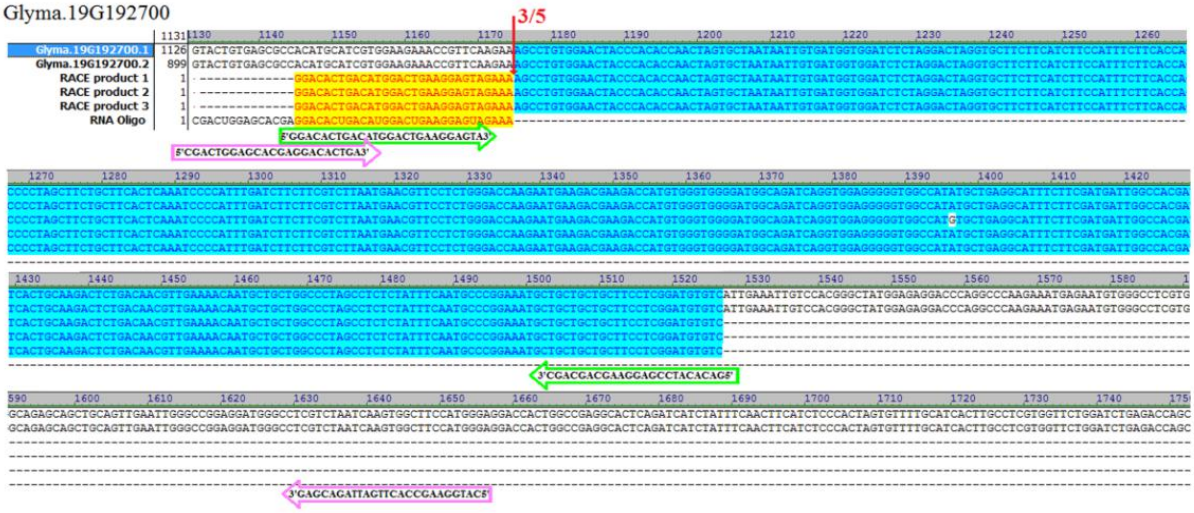

> Glyma.11G208800

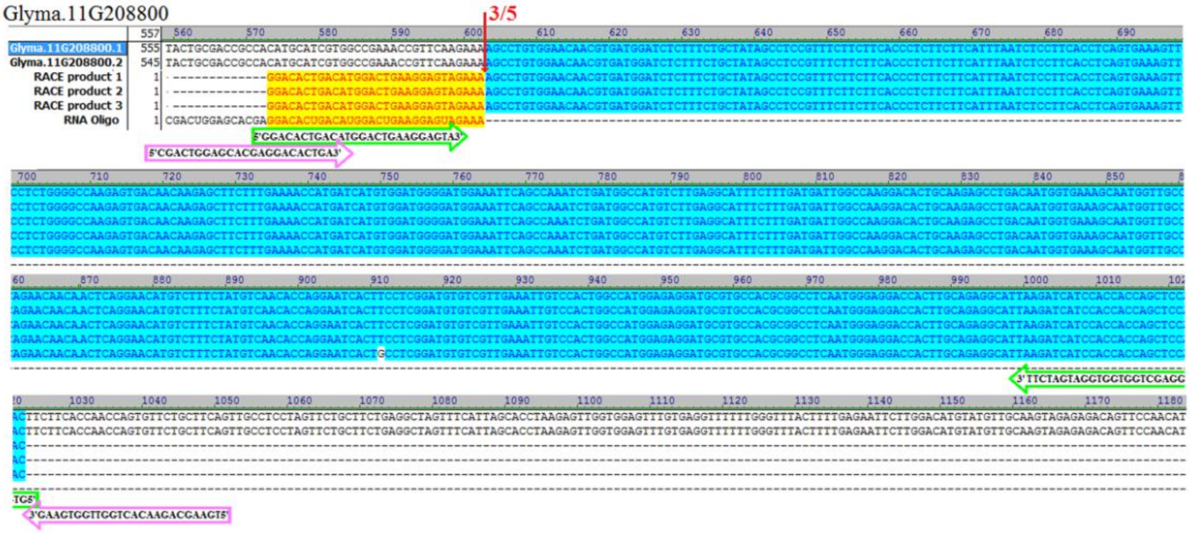

> Glyma.03G021900

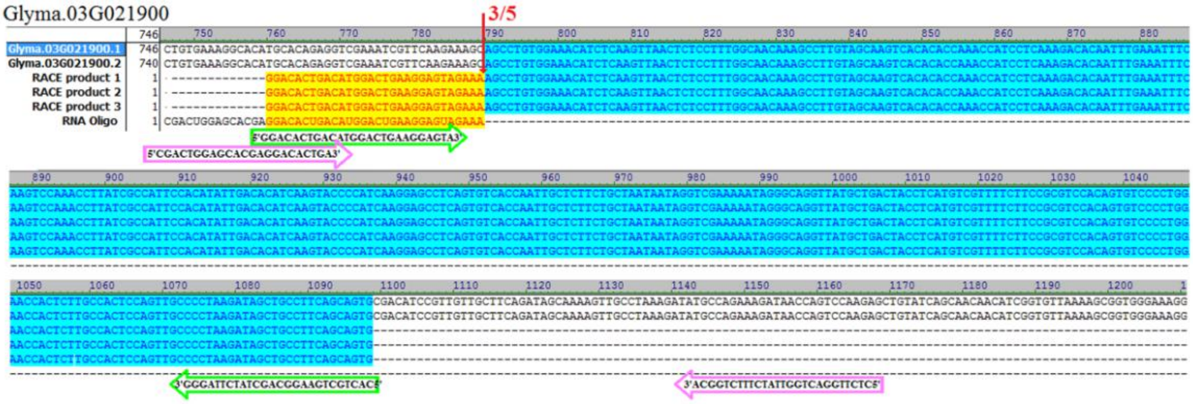

> Glyma.11G008500

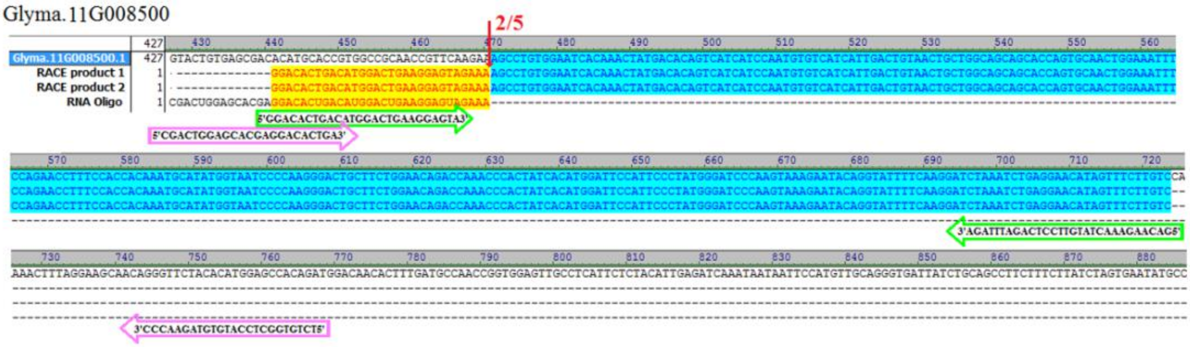

Continuing next

> Glyma.10G067200

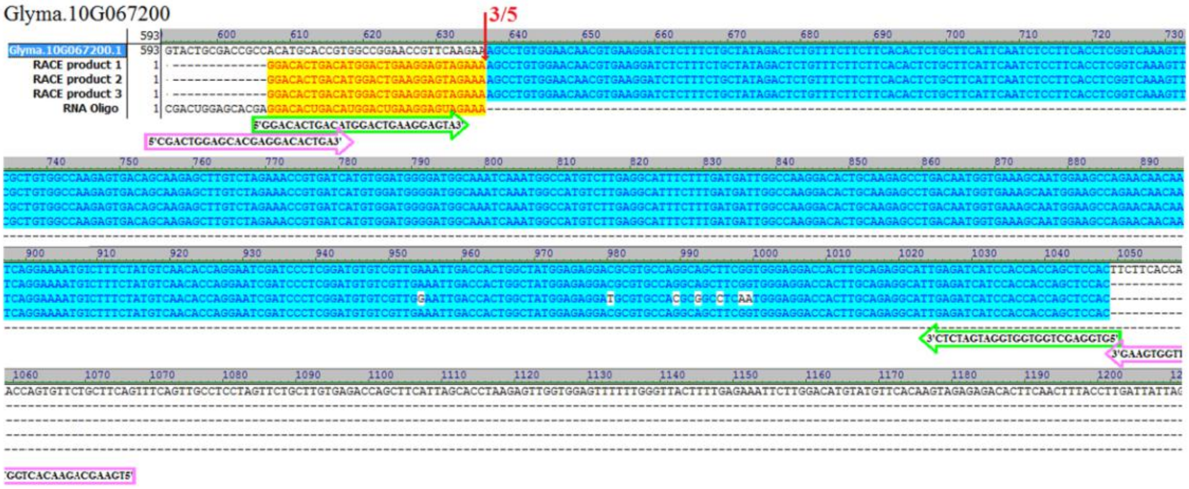

>Glyma.U028600

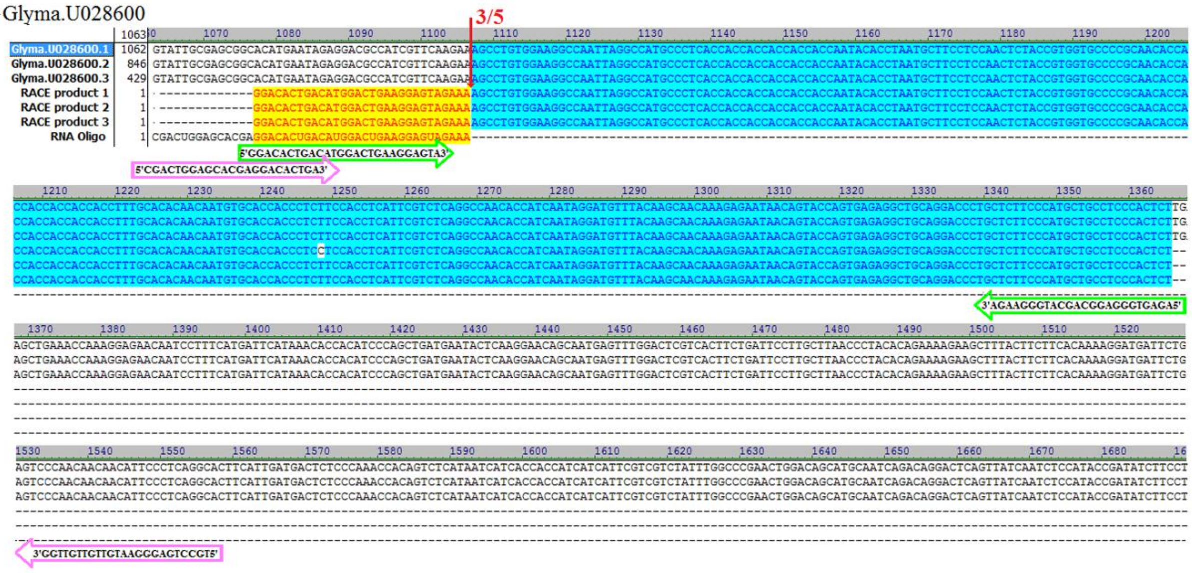

>Glyma.17G232600

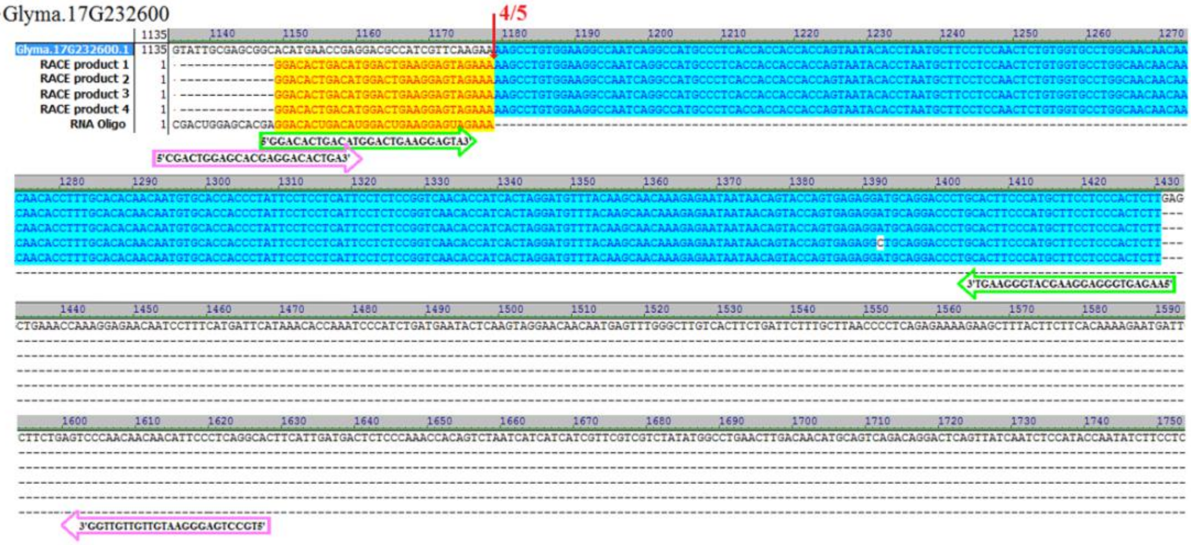

Continuing next

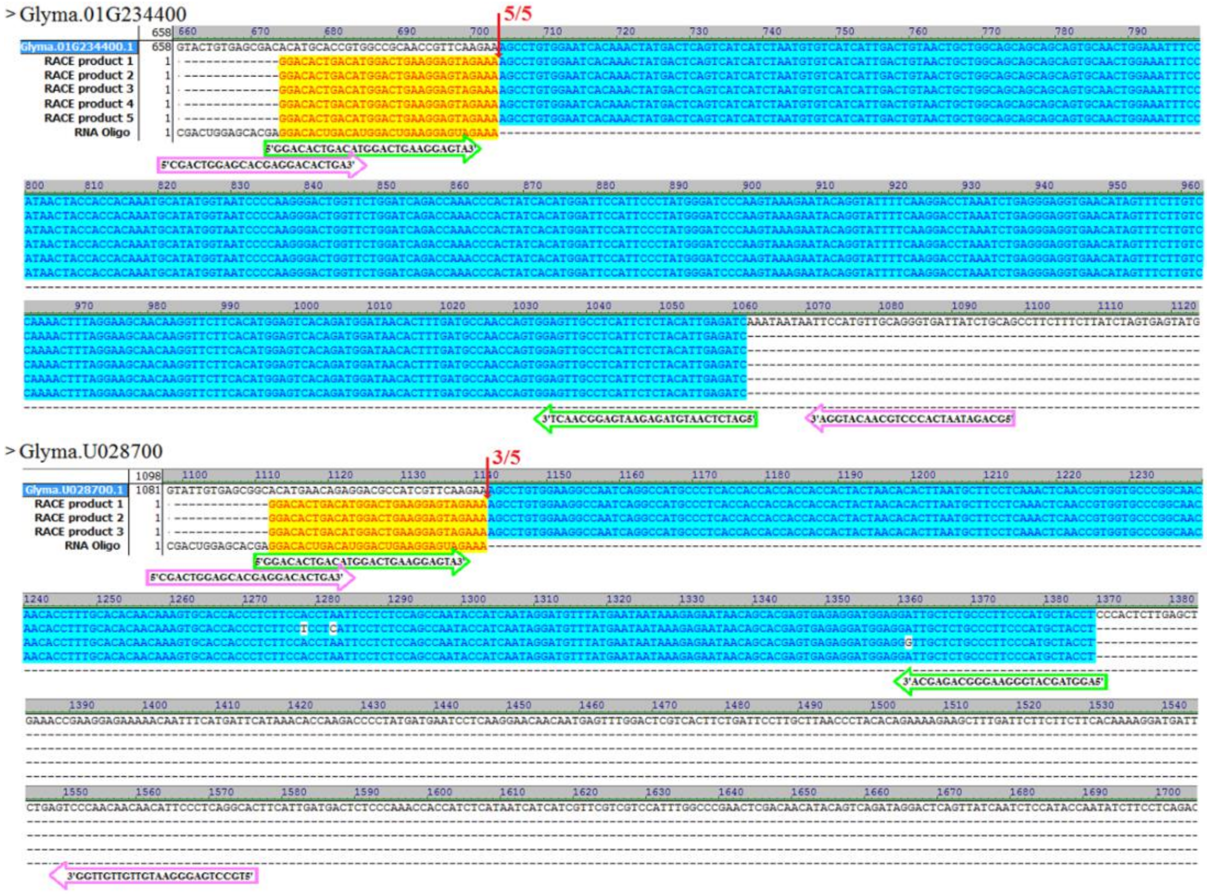

Figure S4. The detailed validation information for cleave sites of gma-miR396s on *GmGRFs* by 5'RACE. Among 26 *GmGRF* family genes (including 57 transcript sequences), 20 *GmGRFs* including 44 transcript sequences (showed in this figure) were confirmed to be cleaved by gma-miR396, their cleave sites were consistently between "CGUUCAAGAA" and "AGCCUGUGGAA"; other 4 *GmGRFs* (Glyma.13G109500, Glyma.17G050200, Glyma.17G232700 and Glyma.07G038400) and Glyma.01G144900.2-4 including 11 transcript sequences have not been validated by 5'RACE, probably because of their lowest expression; and 2 *GmGRFs* (Glyma.11G025900, Glyma.08G265700) were not the target gene of gma-miR396 in prediction.

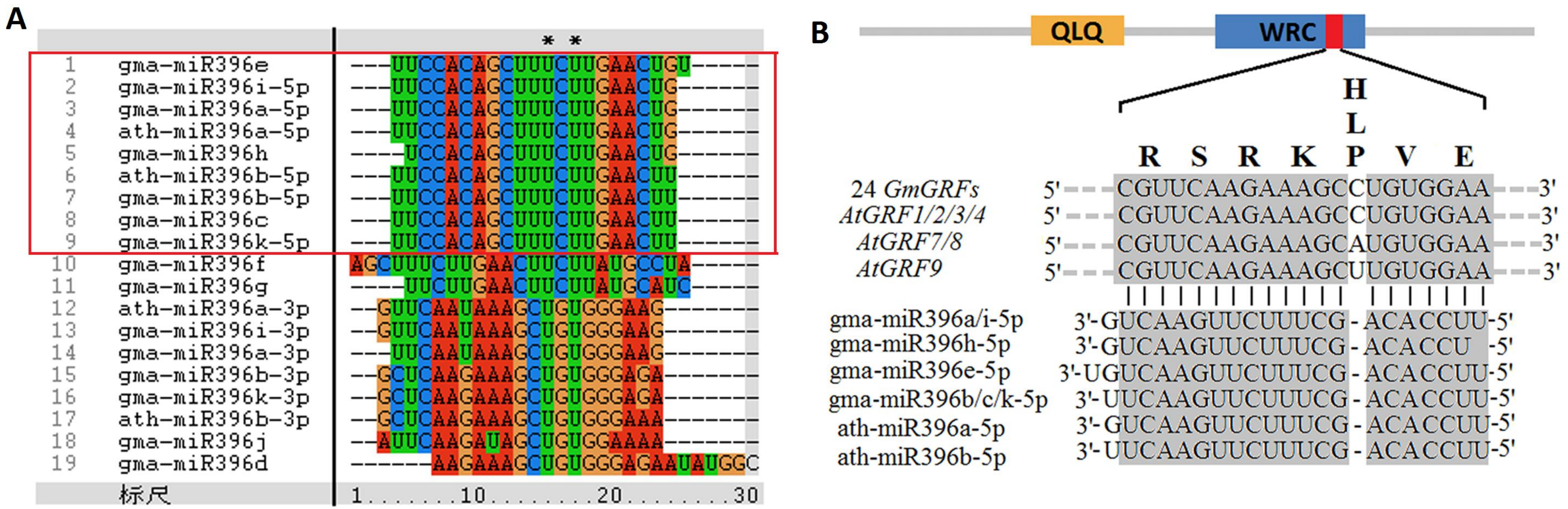

Figure S5. Analysis of the interaction sites between miR396 and *GRF* gene families in soybean and *Arabidopsis*. (A) Sequence alignment among ath-miR396 and gma-miR396 family mature miRNAs. The mature sequences of gma-miR396a/i are same with ath-miR396a; the mature sequences of gma-miR396b/c/k are same with ath-miR396b. Ath-miR396a/b and gma-miR396a/b/c/e/h/i/k which due to 5p miRNAs display high homology. **(B)** Prediction of the interaction between miR396 and *GRF* gene families in soybean and *Arabidopsis.* In soybean, 24 *GmGRFs* were predicted as the targets of gma-miR396a/b/c/e/h/i/k. Actually, we have confirmed 20 *GmGRFs* were the target genes of gma-miR396a/b/c/e/h/i/k on experimental levels in our research. In *Arabidopsis*, it had been confirmed that ath-miR396a and ath-miR396b targeted 7 *AtGRFs* (*AtGRF*1/2/3/4/7/8/9) in precious reports by 5'RACE. In addition, 7 *AtGRFs* (*AtGRF*1/2/3/4/7/8/9) were predicted as the target genes of gma-miR396a/b/c/e/h/i/k in miR396 a/b/c/e/h/i/k-OE transgenic *Arabidopsis*. In addition, gma-miR396a/b/i/k-3p, ath-miR396a/b-3p and gma-miR396d/f/g/j in soybean and *Arabidopsis* can't interact with *GmGRF* and *AtGRF* in prediction. In other words, they can't complement with *GRFs*, so they be omitted in (B).
